# The Chromosomal *parDE2* Toxin–Antitoxin System of *Mycobacterium tuberculosis* H37Rv: Genetic and Functional Characterization

**DOI:** 10.3389/fmicb.2016.00886

**Published:** 2016-06-14

**Authors:** Manish Gupta, Nishtha Nayyar, Meenakshi Chawla, Ramakrishnan Sitaraman, Rakesh Bhatnagar, Nirupama Banerjee

**Affiliations:** ^1^Department of Biotechnology, TERI University, NewDelhi, India; ^2^Molecular and Cell Biology Laboratory, School of Biotechnology, Jawaharlal Nehru UniversityNew Delhi, India; ^3^Institute of Stem Cell Biology and Regenerative Medicine, National Centre for Biological SciencesBangalore, India

**Keywords:** *Mycobacterium tuberculosis*, *ParDE2*, DNA gyrase inhibitor, bacteriostasis, VBNC, stress response, toxin–antitoxin system, selfish genes

## Abstract

*Mycobacterium tuberculosis* H37Rv escapes host-generated stresses by entering a dormant persistent state. Activation of toxin-antitoxin modules is one of the mechanisms known to trigger such a state with low metabolic activity. *M. tuberculosis* harbors a large number of TA systems mostly located within discernible genomic islands. We have investigated the *parDE2* operon of *M. tuberculosis* H37Rv encoding MParE2 toxin and MParD2 antitoxin proteins. The *parDE2* locus was transcriptionally active from growth phase till late stationary phase in *M. tuberculosis*. A functional promoter located upstream of *parD2* GTG start-site was identified by 5′-RACE and *lacZ* reporter assay. The MParD2 protein transcriptionally regulated the P*_parDE2_* promoter by interacting through Arg16 and Ser15 residues located in the N-terminus. In *Escherichia coli*, ectopic expression of MParE2 inhibited growth in early stages, with a drastic reduction in colony forming units. Live-dead analysis revealed that the reduction was not due to cell death alone but due to formation of viable but non-culturable cells (VBNCs) also. The toxic activity of the protein, identified in the C-terminal residues Glu98 and Arg102, was neutralized by the antitoxin MParD2, both *in vivo* and *in vitro*. MParE2 inhibited mycobacterial DNA gyrase and interacted with the GyrB subunit without affecting its ATPase activity. Introduction of *parE2* gene in the heterologous *M. smegmatis* host prevented growth and colony formation by the transformed cells. An *M. smegmatis* strain containing the *parDE2* operon also switched to a non-culturable phenotype in response to oxidative stress. Loss in colony-forming ability of a major part of the MParE2 expressing cells suggests its potential role in dormancy, a cellular strategy for adaptation to environmental stresses. Our study has laid the foundation for future investigations to explore the physiological significance of *parDE2* operon in mycobacterial pathogenesis.

## Introduction

Tuberculosis (TB) continues to be a global health problem due to increasing morbidity and mortality. According to World Health Organization, 9.6 million people developed TB in 2014 and 1.5 million died from the disease^1^. The leading causes of high incidence of *Mycobacterium tuberculosis* (Mtb) infection are the emergence of drug resistant strains, persistence, latency-mediated reactivation of the pathogen and HIV-associated immunodeficiency. *M. tuberculosis* acquires its pathogenicity by virtue of its unique virulence factors that have made it better adapted to persist chronically in hostile niches within the host.

Mycobacteria often embrace a non-replicative persistent, quiescent state, enabling them to evade unfavorable conditions like hypoxia, oxidative stress and acidic pH etc., typically encountered within the host macrophages (Wu et al., 2012). A similar non-replicating state is triggered under stress by the activation of toxin–antitoxin (TA) systems, suggesting that specific TA systems may be involved in adaptation to environmental cues in the host (Lewis, 2007). The TA systems on the one hand protect the bacterial cells from stressful conditions (Ramage et al., 2009), while on the other they initiate programmed cell death for the benefit of the population (Ramage et al., 2009). Genome sequence analysis revealed that the Mtb H37Rv harbors 88 putative TA systems which are mostly located within discernible genomic islands and likely acquired by recent events of horizontal gene transfer. A majority of these are conserved in *M. tuberculosis* complex (MTBC) but absent in other non-pathogenic mycobacteria suggesting a potential contribution to the pathogenic lifestyle of Mtb (Ramage et al., 2009).

The first TA system was originally identified as an extra-chromosomal genetic element involved in post-segregational killing (PSK) of plasmid-free daughter cells to ensure stable maintenance of the plasmid in a population of bacteria (Ogura and Hiraga, 1983). TA systems are widely distributed in both bacteria and archaea (Yamaguchi et al., 2011). Currently known TA systems are classified into three canonical types, majority of them belong to type II family, that is typically a two-gene operon model, encoding an antitoxin protein which binds to and precludes the cognate toxin protein from disrupting an essential cellular function (Gerdes et al., 2005). The ‘addiction’ mechanism behind plasmid inheritance relies upon differential stability of the labile antitoxin protein that is prone to protease degradation, compared to the stable toxin. The antitoxin is degraded in the daughter cells lacking the plasmid, thereby releasing the toxin from the TA complex, which leads to toxin-mediated growth inhibition and/or killing (Melderen et al., 1994; Lehnherr and Yarmolinsky, 1995). From an evolutionary point of view, TA systems are ‘selfish’ genetic elements that ensure their own vertical transmission by post-segregational host killing for reasons of adaptive advantage (Van Melderen and De Bast, 2009). This is analogous to maintenance of type II restriction-modification (R-M) systems, where post-segregational host killing ensures inheritance of the R-M system as a unit (Naito et al., 1995; Kobayashi, 2001).

The post-genomic era has revealed that TA modules are diverse and ubiquitous in prokaryotic genomes as a consequence of horizontal gene transfer. TA loci along with a vast collection of transposons, phages and integrative and conjugative elements constitute the ‘mobilome’ of bacteria. The chromosomal toxins which are either DNA replication or protein synthesis inhibitor belong to nine phylogenetically distinct classes (Hayes and Van Melderen, 2011). The most well-investigated, genome-encoded TA systems have come from the pioneering work in *Escherichia coli* (Gerdes et al., 2005). A few of these modules are triggered under stress leading either to cell stasis or programmed cell death (Ramage et al., 2009). Thus, the role of TA systems, especially those borne on the nucleoid, as agents of adaptation and evolution in prokaryotes is likely to be significant.

The prototypic CcdB and ParE proteins of *E. coli* are the toxin components of the CcdAB and ParDE TA systems, encoded by broad host range F and Rk2 plasmids, respectively (Bernard and Couturier, 1992; Jiang et al., 2002). Notwithstanding low similarity at amino acid level, both CcdB and ParE block DNA replication and transcription by inhibiting DNA gyrase in a manner similar to that of fluoroquinolone antibiotics (Jiang et al., 2002), although they recognize different regions of DNA gyrase (Yuan et al., 2010). ParE toxins share considerable protein sequence homology with the toxin components of the RelBE family that poison the translation machinery of a cell (Pandey and Gerdes, 2005). The RK2-encoded negatively charged ParD antitoxin dimer was shown to neutralize the positively charged ParE toxin by forming a stable D_2_E_2_ tetramer complex (Roberts et al., 1993; Johnson et al., 1996; Dalton and Crosson, 2010). The N-terminal half of ParD adopts a ribbon-helix-helix (RHH) fold and auto-regulates the *parDE* operon negatively (Oberer et al., 2007). Genome-encoded *parDE* TA systems are widespread within the γ-proteobacterium class (Anantharaman and Aravind, 2003). *Caulobacter crescentus*, an α-proteobacterium, encodes three functional *parDE* modules that are differentially and independently regulated under various stress conditions (Fiebig et al., 2010). Three homologs of RK2 encoded *parDE* loci, reported in the Gram-negative rod *Vibrio cholerae* superintegron, were found to be active in *E. coli* also (Yuan et al., 2011).

The topological stress generated during DNA replication and transcription is relieved by DNA gyrase (a type II topoisomerase) via a strand breakage and re-joining process in an energy-dependant manner, thereby maintaining the correct level of chromosomal super-helicity for apt functioning of essential cellular processes (Reece et al., 1991; Schoeﬄer and Berger, 2008). The ParE2 of *V. cholera*, like the CcdB toxin and quinolone antibiotics, targets the GyrA subunit of the DNA gyrase holoenzyme (an A_2_B_2_ tetramer) and stalls the DNA-gyrase cleavage complex (Loris et al., 1999; Yuan et al., 2010).

Based on the DNA sequence homology, two *parDE* family members, Rv1960c-Rv1959c (*parDE1*) and Rv2142A-Rv2142c (*parDE2*) were identified in *M. tuberculosis* H37Rv genome (Ramage et al., 2009). This study provides a comprehensive overview of the *parDE2* locus of *M. tuberculosis* H37Rv. We report genetic organization and transcriptional regulation of the operon, and describe biochemical characteristics of the MParD2 and MParE2 proteins. We also investigate its potential involvement in adaptation to environmental stresses using heterologous hosts- *E. coli* and *M. smegmatis*.

## Experimental Procedures

### Bacterial Strain, Plasmid, and Culture Conditions

The plasmids and bacterial strains used in this study are listed in **Table 1**. *E. coli* strains used for cloning were grown under standard conditions in Luria-Bertani (LB) medium (Difco). *M. tuberculosis* and *M. smegmatis* strains were grown in Middlebrook 7H10 agar (Difco) containing 10% (v/v) oleic acid, albumin, dextrose and catalase (OADC; Becton Dickinson) enrichment and 0.5% (v/v) glycerol, or in 7H9 broth (Difco) supplemented with 10% (v/v) albumin, dextrose and catalase (ADC; Becton Dickinson), 0.2% (v/v) glycerol and 0.05% (v/v) Tween 80 (Chandra et al., 2010). Unless indicated otherwise, all cultures were grown at 37°C, with shaking at 180 rpm.

**Table 1:** Bacterial strains and plasmids used in this study.

	Relevant characteristic(s)	Source
**Plasmids**		
pGEM-T Easy	Amp^r^; PCR TA-cloning vector	Promega
pMV261	kan^r^; *E. coli*–mycobacterial shuttle vector	Stover et al., 1991
pYUB1062	Hyg^r^; *E. coli*–mycobacterial shuttle vector	Wang et al., 2010
pET28(a)	kan^r^, T7 promoter; *E. coli* expression vector	Novagen
pBAD/HisA	Amp^r^, *ara*BAD promoter; *E. coli* expression vector	Invitrogen
pGEX4Tl	Amp^r^, *tac* promoter; *E. coli* expression vector	GE Healthcare
pMSl	pMV261 containing promoter-less *lacZ*	This study
pMS2	pMV261 containing P*_363parD2_*-*lacZ*	This study
pMS3	pMS2 containing P*_groEL_*-*parD2*	This study
pMS4	pMS2 containing P*_groEL_*-*parDE2*	This study
pMS5	pYUB1062 containing 318-bp *parE2*	This study
pMS6	pYUB1062 containing 258-bp *parE2Δ10* amino acids	This study
pMS7	pYUB1062 containing 530-bp *parDE2* together	This study
pMS8	pMV261 containing *parDE2* operon	This study
pMS9	pMV261 containing *parDE2A10* operon	This study
pMSlO	pMV261 containing *parD2*	This study
pMSll	pMV261 containing *parDE2*	This study
pECl	pET28(a) containing 2,517-bp *M. tuberculosis gyrA*	This study
pEC2	pET28(a) containing 2,145-bp *M. tuberculosis gyrB*	This study
pEC3	pBAD/HisA containing 216-bp *parD2*	This study
pEC4	pBAD/HisA containing 318-bp *parE2*	This study
pEC5	pBAD/HisA containing 530-bp *parDE2*	This study
pEC6	pBAD/HisA *containing parE2Δ10* amino acids	This study
pEC7	pBAD/HisA containing *parE2^P92A^* mutant	This study
pEC8	pBAD/HisA containing *parE2^E96A^* mutant	This study
pEC9	pBAD/HisA containing *parE2 ^E98A^* mutant	This study
pECIO	pBAD/HisA containing *parE2^I99A^* mutant	This study
pECll	pBAD/HisA containing *parE2^S100A^* mutant	This study
pEC12	pBAD/HisA containing *parE2^R102A^* mutant	This study
pEC13	pBAD/HisA containing *parD2^L8A^* mutant	This study
pEC14	pBAD/HisA containing *parD2^V 11A^* mutant	This study
pEC15	pBAD/HisA containing *parD2^S15A^* mutant	This study
pEC16	pBAD/HisA containing *parD2^R16A^* mutant	This study
pEC17	pBAD/HisA containing *parD2^E21A^* mutant	This study
pEC18	pGEX4T1 containing 216-bp *parD2*	This study
**Bacterial Strains**		
*M. tuberculosis* H37Rv	Wild type	ATCC
*M. smegmatis* mc^2^ 155	*ept-1*, efficient plasmid transformation mutant of mc^2^6	ATCC
*M. smegmatis* mc^2^ 4517	*M. smegmatis* mc^2^ 155 with T7 RNA polymerase	Wang et al., 2010
*E. coli* TOP 10	*recAlaraD139*Δ*(ara-leu)7697 galU galK rpsL(Str^r^)*	Invitrogen
*E. coli* BL21 (DE3)	F^-^ *ompT gal dcm Ion hsdS_B_(r_B_^-^ m_B_^-^)*λ (DE3)	Novagen
*E. coli* Rosetta^TM^ (DE3) pLysS	F^-^ *ompT hsdS_B_(R_B_*^-^ *m_B_*^-^*) gal dcm*λ(DE3 *[lacI lacUV5-T7 gene 1 ind1 sam 7 nin 5*]) pLysSRARE (Cam^r^)	Novagen
MS1	*M. smegmatis* mc^2^ 155 transformed with pMS1	This study
MS2	*M. smegmatis* mc^2^ 155 transformed with pMS2	This study
MS3	*M. smegmatis* mc^2^ 155 transformed with pMS3	This study
MS4	*M. smegmatis* mc^2^ 155 transformed with pMS4	This study
MS5	*M. smegmatis* mc^2^ 4517 transformed with pMS5	This study
MS6	*M. smegmatis* mc^2^ 4517 transformed with pMS6	This study
MS7	*M. smegmatis* mc^2^ 4517 transformed with pMS7	This study
MS8	*M. smegmatis* mc^2^ 155 transformed with pMS8	This study
MS9	*M. smegmatis* mc^2^ 155 transformed with pMS9	This study
MS10	*M. smegmatis* mc^2^ 155 transformed with pMS10	This study
MS11	*M. smegmatis* mc^2^ 155 transformed with pMS11	This study
MS 12	*M. smegmatis* mc^2^ 155 transformed with pMV261	This study
EC1	*E. coli* BL21(DE3) transformed with pEC1	This study
EC2	*E. coli* BL21(DE3) transformed with pEC2	This study
EC3	*E. coli* TOP10 transformed with pEC3	This study
EC4	*E. coli* TOP10 transformed with pEC4	This study
EC5	*E. coli* TOP10 transformed with pEC5	This study
EC6	*E. coli* TOP10 transformed with pEC6	This study
EC7	*E. coli* TOP10 transformed with pEC7	This study
EC8	*E. coli* TOP10 transformed with pEC8	This study
EC9	*E. coli* TOP10 transformed with pEC9	This study
EC10	*E. coli* TOP10 transformed with pEC10	This study
EC11	*E. coli* TOP10 transformed with pEC11	This study
EC12	*E. coli* TOP10 transformed with pEC12	This study
EC13	*E. coli* TOP10 transformed with pEC13	This study
EC14	*E. coli* TOP10 transformed with pEC14	This study
EC15	*E. coli* TOP10 transformed with pEC15	This study
EC16	*E. coli* TOP10 transformed with pEC16	This study
EC17	*E. coli* TOP10 transformed with pEC17	This study
EC18	*E. coli* BL21(DE3) transformed with pEC18	This study

### Protein Expression and Purification

The DNA encoding *gyrA*, *gyrB, parD2, parE2*, and *parDE2* loci of *M. tuberculosis* H37Rv were amplified by PCR and cloned in suitable expression vectors (**Table 1**), with His_6_ tag in N-termini. Briefly, one liter culture of recombinant *E. coli* strains EC1, EC2, and EC3 were grown at 37°C in LB supplemented with appropriate antibiotics to an A_600_ of ~0.6. Protein expression was induced with 1mM isopropyl-β-D-thiogalactopyranoside (IPTG) or 0.2% L-arabinose, and incubation was continued for an additional 4 h at 37°C for MParE2 and MParD2; and 8 h at 30°C for GyrA and GyrB, respectively. The cells were lysed by sonication and the His_6_-tagged proteins were purified using Ni-NTA affinity chromatography (Qiagen). The column was equilibrated with binding buffer B (50 mM Tris-Cl, pH 8.0, 300 mM NaCl, 20 mM imidazole, 5% glycerol) and the cell lysate was loaded. After washing with buffer W (buffer B containing 50 mM imidazole), recombinant proteins were eluted from the column with a 20-ml linear imidazole gradient (20–500 mM imidazole). Fractions containing pure protein (purity >90% estimated by SDS-PAGE) were pooled and dialyzed at 4°C overnight, in protein storage buffer (buffer B containing 2 mM βME, 20% glycerol).

The MParD2 protein was further purified by heparin-sepharose CL-6B affinity chromatography (GE Healthcare). The column was equilibrated in 25 mM phosphate buffer (pH 7.4) containing 0.1 M NaCl and the same buffer was used for MParD2 binding and washing. Protein was eluted using a linear 0.1 M to 1.5 M NaCl gradient and 0.5 ml fractions were collected. The fractions eluting at 0.4 to 0.7 M NaCl were pooled and dialyzed in potassium glutamate buffer R (40 mM Tris–HCl pH 7.4, 100 mM potassium glutamate, 10 mM MgCl_2_, 25 mM KCl, 4 mM DTT and 10% glycerol) and checked for DNase contamination by incubation with supercoiled plasmid DNA. The MParE2 protein was purified from the inclusion bodies by Ni-NTA affinity chromatography under denaturing conditions as described before (Singh et al., 2004). The fractions were analyzed by 12% SDS–PAGE. The fractions containing the recombinant MParE2 were pooled, concentrated, and dialyzed against protein storage buffer (see above) and stored at 4°C. For preparing MParE2 without the His_6_-tag, an N-terminal thrombin cleavage site was inserted before the start codon in the *parE2* gene and sub-cloned in the pBAD/HisA expression vector using NcoI and HindIII sites.

For surface plasmon resonance (SPR) experiments the proteins were purified further. The recombinant GyrA and GyrB proteins obtained from Ni-NTA affinity chromatography were further purified to >99% purity by Superdex-200 pg column. Fractions were pooled and dialyzed in 1XHBS-N buffer (10 mm HEPES pH 7.4, 150 mm NaCl). The MParE2 purified by Ni-NTA under denaturing conditions was concentrated and dialyzed in 1X cleavage buffer (50 mM Tris-HCl pH 8.0, 10 mM CaCl_2_ and 100 mM NaCl). The N-terminal His_6_-tag was removed by using RECOMT Thrombin Cleavage Kit (Sigma) according to the manufactures protocol. The flow-through fraction was subjected to Ni-NTA chromatography again to remove any residual His_6_-MParE2. Unbound fraction was collected and dialyzed in 1XHBS-N buffer for further use.

### Deletion and Point Mutations in MParE2 and MParD2

The MParE2 variant with C-terminal deletion of 10 amino acid residues, MParE2Δ10 (1–95 residues) was generated by polymerase chain reaction using the wild type *parE2* gene as the template and deletion primers (Supplementary Figure S3). In MParE2, Pro92, Glu96, Glu98, Ile99, Ser100, and Arg102 residues were substituted individually with alanine, using appropriate primers (Supplementary Figure S3). In MParD2 the amino acid residues Leu8, Val11, Ser15, Arg16, and Glu21 in the N-terminus were substituted individually by alanine using suitable primers (Supplementary Figure S3). All the protein variants were cloned in the pBAD/HisA vector and transformed in *E. coli* TOP10 cells. Growth profile and cellular viability of the strains (EC6 to EC17) was monitored after L-arabinose induction as described above. The MParD2 variants were purified similar to wild-type MParD2 protein.

### RNA Extraction and Semi-Quantitative Reverse Transcriptase PCR

Cultures of *M. tuberculosis* were grown in 7H9 medium, cells were harvested at different time points (10, 20, and 30 days), and cells were lysed using TRIzol (Invitrogen) with ceramic beads in a MagNA-Lyser (Roche). RNA was extracted with chloroform/isoamyl alcohol. Contaminating DNA was removed by digestion with DNase-I according to the manufacturer’s instructions (Promega), and confirmed by PCR using *parDE2* operon-specific primers. For RT-PCR, the Qiagen one-step Reverse Transcriptase kit was used according to the manufacturer’s instructions (Qiagen). RNA was reverse transcribed and the cDNA was amplified with *parDE2* operon-specific primers over 28 cycles of PCR. The secreted antigen ESAT-6 gene (*esxA*) was used as positive control.

### Real-Time PCR

The transcription profile of three commonly used reference genes (Rocha et al., 2015), *rrsG*, *idnT*, and *rpoD* encoding the 16S rRNA, the L-idonate/5-ketogluconate/gluconate transporter and the sigma 70 (sigma D) subunit, respectively, were quantified by qPCR in EC4 and vector control *E. coli* strains after induction with L-arabinose for 4 h. Primers were designed using GenScript Real-time PCR (TaqMan) Primer Design tool and synthesized by Sigma–Aldrich. Genomic DNA free, total RNA was obtained as described above. Real-time PCR was performed with a Stratagene Mx3000P (Agilent Technologies) using the High-Capacity cDNA Reverse Transcription Kit and SYBR Select Master Mix (Applied Biosystems) under conditions as mentioned earlier (Lee et al., 2010). Data was analyzed using Stratagene MxPro QPCR software version 4.10.

### Fusion of Mtb *parD2* Upstream Sequence with *lacZ* Reporter Gene and Evaluation of Promoter Activity

The 363-bp upstream region preceding the *parD2* start codon (P*_363parD2_*) of *M. tuberculosis* H37Rv was PCR-amplified and fused to a promoter-less *lacZ* reporter gene (3,067 bp) in pSK6 plasmid (Kant et al., 2009). The 363-bp *lacZ* fusion fragment was subsequently cloned in an *E. coli*-*Mycobacterium* shuttle vector pMV261, using NotI and DraI sites, forming pMS2 (Supplementary Figure S2). A promoter-less *lacZ* gene cloned in pMV261 (pMS1) was used as negative control. For *parDE2* transcriptional regulation studies, *parD2* or *parDE2* coding sequences were placed downstream of the *groEL* promoter of pMS2, at restriction sites EcoR1 and HindIII, to form pMS3 and pMS4, respectively (Supplementary Figure S2). All these recombinant constructs were electroporated into *M. smegmatis* mc^2^ 155.

β-galactosidase activity in all the *M. smegmatis* recombinants was determined colorimetrically using *O*-nitrophenyl-β-D-galactopyranoside (ONPG; Sigma), as described earlier (Miller, 1972; Dellagostin et al., 1995). Absorbance was measured at 420 nm on a Cecil Aquarius CE 7500 Spectrophotometer. One unit of β-galactosidase is the amount of enzyme producing 1 nmol of *O*-nitrophenol per min at 30°C. Each experiment was performed thrice in triplicate.

### Mapping the 5′ End of the *parDE2* Transcript

Total RNA was extracted from *M. tuberculosis* H37Rv as before and after DNaseI (Promega) treatment, 5′ Rapid amplification of cDNA ends (RACE) was performed according to manufacturer’s protocol (Invitrogen). The RT reaction was performed with D2-GSPI (5′-TCATCCGAGCCGGGCGCGGATCCG-3′) andthen PCR Amplified with the Abridged Anchor Primer (AAP, Invitrogen) and D2-GSPII (5′-GGATCCGCTTGTCGA AGTCATCAA-3′). The final PCR product was cloned in pGEM-T Easy TA cloning vector and sequenced using T7 and SP6 promoter-specific universal primers.

### Electrophoretic Mobility Shift Assay (EMSA)

His_6_-tagged MParD2 protein was purified as described above and dialysed against 1X EMSA binding buffer (10 mM Tris-HCl pH 8.0, 1 mM DTT, 2.5 mM MgCl_2_, 40 mM KCl, 0.5 μg ul^-1^ dI:dC, 5% glycerol). The 200 bp upstream of the *parD2* start site, P*_200parD2_* was PCR amplified, end-labeled with [γ-^32^P]-ATP using T4 polynucleotide kinase (Promega) and purified using G-25 MicroSpin column (GE Healthcare). The MParD2 or the variant proteins were incubated with 20,000 cpm radiolabelled P*_200parD2_* in 1X EMSA binding buffer at room temperature for 20 min, in a reaction mix of 25 μl. Non-denaturing DNA loading dye was added to the reaction mixture and electrophoresed on 5% Native-PAGE gel in 0.5X TBE buffer at 4°C at 60 V till bromophenol blue reached the bottom. The gel was dried and exposed to a phosphorimager screen (Amersham Biosciences) overnight and scanned in a Typhoon Imager (GE Healthcare).

### ANS Binding Assay

8-Anilinonaphthalene-1-sulfonic acid (ANS) was dissolved in 100 mM phosphate buffer to a final concentration of 500 μM. ANS fluorescence measurements as a function of protein folding were performed at 25°C at an excitation wavelength of 350 nm and the emission spectra was recorded from 360 to 600 nm (Taylor and Kneale, 2009). One μM MParD2 or variant proteins were mixed with 5 μM of ANS, to get stable fluorescence emission. Subsequently, 0.12 μM P*_200parD2_* DNA was added to the MParD2-ANS mix and changes in the fluorescent intensities at emission maxima (480 nm) were determined. Control experiments lacking either the MParD2 antitoxin or the P*_200parD2_* DNA were also included. The fluorescent intensity emitted by 5 μM ANS in 100 mM phosphate buffer was set as the baseline and hence subtracted from all the sample readings.

### Growth of *E. coli* and *M. smegmatis* Strains Containing *parE2* and *parDE2*

*Escherichia coli* strains were grown in LB medium containing 100 μg ml^-1^ of ampicillin and 50 μg ml^-1^ of streptomycin at 37°C. Freshly inoculated cultures were grown for 2 h (MParE2 expressing cultures were grown for 6 h as they grow slowly), till OD_600_ ~0.5, and induced with 0.2% L-Arabinose (final concentration). Samples were aseptically withdrawn at regular intervals and growth was monitored by measuring OD_600_ and colony forming units (CFU) by serial dilution and plating on LB agar plates containing suitable antibiotics. In another experiment the incubation was terminated after 4 h, cells were washed with LB medium and re-suspended in same volume of fresh LB medium containing appropriate antibiotics and 0.2% D-glucose to stop toxin expression and incubation was continued. The recombinant *E. coli* strains were also induced in different growth phases corresponding to OD_600_ of 0.33, 0.78, 1.12, and 1.61. MParE2 expression and toxicity was examined by western blotting and CFU counts.

*Mycobacterium smegmatis* recombinant strains were generated by electroporating wt-*parE2* and *parDE2* DNA cloned in the *E. coli*-mycobacterial shuttle vector pYUB1062, into electro-competent *M. smegmatis* mc^2^ 4517 cells (using 10% glycerol) and selecting transformants on 7H10 agar containing OADC supplement and 50 μg ml^-1^ hygromycin.

### Measurement of Viability by SYBR Green I/Propidium Iodide Assay

To assay cell viability of MParE2 expressing *E. coli* cells, the EC4 and EC5 strains were grown to log phase (A600 ~0.5) and protein expression was induced with 0.2% L-arabinose for 4 h at 37°C. Cells were harvested after 2, 4, 8, 12, and 20 h post induction; washed and diluted in 1X PBS (pH 7.4). The assays were performed in 96 well microplates (Feng et al., 2014b). For staining, 10 μl of SYBR Green I (10,000X stock; Lumiprobe) was mixed with 30 μl of propidium iodide (PI; 20 mM; Sigma) and the mixture was added to 960 μl of sterile 1X PBS and vortexed. Ten microliters of freshly prepared staining mixture were added to each well containing ~1 × 10^6^ cells. The excitation/emission maxima are 485/535 nm for SYBR Green I (Green emission) and 485/635 nm for PI (Red emission). The Green (G) and Red (R) fluorescence intensities were measured using SpectraMax^®^ M3 Multi-Mode Microplate Reader, Molecular Devices; followed by data acquisition and analysis using SoftMax Pro 6.3 software. For quantitative estimation, a standard curve was prepared by mixing 100% live and 100% dead (Isopropanol treated) *E. coli* cells in different proportions (viz. 10:0, 8:2, 5:5, 2:8, 0:10) and the resulting G/R fluorescence ratio was plotted. The association between percentage cell viability and corresponding green/red fluorescence was obtained from a regression equation and curve following the least-square fitting method. Sparfloxacin (0.1 μg ml^-1^) treated *E. coli* cells containing pBAD/HisA were used as a positive control.

### Scanning Electron Microscopy (SEM)

Surface topography of the bacterial cells was evaluated by SEM analysis as described earlier (Singh and Mukhopadhyay, 2011). EC4 and EC5 cells were induced and harvested by centrifugation, washed three times with 1X PBS (pH 7.2) and fixed with 2.5% (v/v) glutaraldehyde in 0.1 M phosphate buffer (pH 7.2), at 4°C, overnight. The fixed cells were washed three times with 0.1 M phosphate buffer (pH 7.2) and dehydrated in a series of graded ethanol solutions and dried with hexamethyldisilazane (HMDS) on cover slips and mounted on the SEM stub. Samples were viewed on a Carl Zeiss EVO 40 scanning electron microscope at 20 KeV.

### Interaction of MParE2 Toxin with MParD2 Antitoxin

GST-tagged MParD2 and GST tag alone (as negative-bait control) were purified as follows: IPTG-induced 500 ml EC18 culture was harvested and lysed by sonication in 20 ml binding Buffer-A (50 mM Tris-Cl pH 7.5, 50 mM NaCl, 5% glycerol and 1 mM EDTA). The lysate was loaded on a glutathione sepharose affinity column (GE Life Sciences) and eluted in Buffer-A containing reduced glutathione. The purified GST-D2 or GST proteins (after dialysis against buffer A) were mixed with partially purified His6X-MParE2 in Buffer A and incubated overnight at 4°C for interaction. The mixture was loaded on a glutathione sepharose column, washed with Buffer-A containing 2 mM reduced glutathione and eluted in Buffer-B (Buffer-A containing 300 mM NaCl and 20 mM reduced glutathione). The eluted fractions were concentrated by Amicon-ULTRA (MWCO 3KDa) filtration. Partially purified His6X-Domain 4 (PA-D4) of the protective antigen of *Bacillus anthracis* (Manish et al., 2013) was used as non-specific protein-prey control. Protein samples were resolved using SDS-PAGE and immunoblotting was performed using anti-GST (Santa Cruz biotech) and anti-His (Sigma) monoclonal antibodies.

### Purification and Reconstitution of Mycobacterial DNA Gyrase A_2_B_2_ Holoenzyme

Purified Mtb GyrA and GyrB subunits were mixed in a molar ratio of 1:1.5 and dialysed against potassium glutamate Buffer R. The reconstituted mixture was applied to a Superdex-200 pg column (16/600 HR; Amersham Pharmacia Biotech) pre-equilibrated with Buffer R and resolved using an AKTA FPLC system (Amersham Pharmacia Biotech). The molecular weights of the proteins were estimated by Blue Native-PAGE and SDS-PAGE with appropriate standards (data not shown).

### DNA Supercoiling Assays

The DNA gyrase supercoiling activity was determined by measuring the conversion of relaxed plasmid pBR322 DNA to the supercoiled form. The relaxed pBR322 DNA was prepared from the supercoiled form (Inspiralis Ltd.) by treatment with wheat germ topoisomerase-I as described by the manufacturer (Promega). The supercoiling reactions were carried out as follows: A standard reaction mixture of 30 μl contained 30 mM Tris-Cl (pH 7.4), 25 mM KCl, 10 mM MgCl_2_, 2 mM spermidine, 3 mM DTT, 0.1 mg ml^-1^ yeast tRNA, 0.42 mg ml^-1^ BSA, 120 mM potassium glutamate, 1 mM ATP, nuclease-free water, 1 μg of relaxed pBR322 plasmid DNA as substrate and variable amounts of Mtb DNA gyrase holoenzyme. The reaction mixture was incubated at 37°C for 1 h, and terminated by the addition of 0.1% SDS and 100 μg ml^-1^proteinase K; followed by further incubation for 30 min at 37°C. The reaction mixture was subjected to electrophoresis in a 0.8% agarose gel in 0.5X TBE buffer (Tris-borate-EDTA, pH 8.3) overnight at 25 V, and the gel was stained with ethidium bromide (1 μg ml^-1^). One unit of enzyme activity was defined as the amount of DNA gyrase that converted 1 μg of relaxed pBR322 to the supercoiled form in 1 h at 37°C.

For inhibition and reversal of DNA gyrase activity, supercoiling assay was carried out as above in the presence of toxin or toxin and antitoxin together. For each reaction 1 unit of DNA gyrase holoenzyme was used. For inhibition, 1.3, 2.6, and 5.3 μM MParE2 was mixed with 1 unit of DNA gyrase individually and incubated for 30 min at RT before commencing the supercoiling assay. To reverse the inhibitory effect of the toxin, 5.3 μM MParE2 was mixed with 3.6, 7.2, and 10.9 μM MParD2, prior to addition of DNA Gyrase. Sparfloxacin (a fluoroquinolone) at concentrations of 2 and 5 μg ml^-1^ was used as positive control for gyrase inhibition in the DNA gyrase assay. The ATPase activity of the GyrB protein was tested as described earlier (Anderle et al., 2008).

### Interaction of MParE2 with DNA Gyrase Subunits

To examine the interaction of MParE2 with DNA gyrase subunits, isothermal titration calorimetry (ITC) was performed at 25°C using the MicroCal iTC200 system (GE Healthcare). For all isotherms, the sample cell was filled with 300 μl of 70 μM GyrA/GyrB and the syringe was loaded with 700 μM ParE2 toxin. Each binding isotherm was obtained from 20 injections of ParE2 toxin (2 μl each) at 25°C, with a spacing of 150 s to allow for the re-attainment of a differential power baseline of 9.0 μcal s^-1^. The stirring speed and reference power were 1000 rpm and 5 μcal s^-1^, respectively. Binding isotherms were integrated and analyzed using Origin v7.0 software (MicroCal).

SPR experiments were performed using BIAcore^®^ T200 (GE Healthcare). GyrA and GyrB subunits were captured via their N-terminal His_6_-tags by monoclonal anti-His antibody immobilized on a CM4 sensor chip (carboxymethylated dextran chips, GE Healthcare) at a surface density of 2,500 RU, using amine-coupling chemistry. The kinetic studies were setup by passing different analyte concentrations ranging from 1.1 to 18 μM at 20 μl min^-1^ with a dissociation time of 600 s. Association time was kept constant at 180 s. Regeneration was subsequently performed by injecting 40 μl of regeneration buffer (10 mM glycine pH 2.1) at 10 μl min^-1^. Binding of MParE2 to the GyrA and GyrB subunits was analyzed by the BIAcore^®^ evaluation software. Sparfloxacin binding to immobilized GyrA subunit was used as a positive control.

### Exposure of *M. smegmatis* Containing the *parDE2* Operon to Stress Conditions

To examine the role of *parDE2* operon in *M. smegmatis*, recombinant strains were subjected to various stress conditions and viability of the cultures was determined. MS8 (containing wt-*parDE2* operon), MS9 (containing C′-terminal truncated *parDE2* operon) and MS12 (pMV261 vector control) strains were inoculated into 7H9 broth with supplements and 40 μg ml^-1^ kanamycin, and grown at 37°C with shaking at 180 rpm. Aliquots were withdrawn at different time points, washed with fresh 7H9 broth and subjected to different stress conditions. CFU counts were determined before and after stress treatment by plating serial dilutions on 7H10 agar plates containing 40 μg ml^-1^kanamycin and incubated at 37°C for 3–4 days.

For exposure to hypoxia, mycobacterial cultures were grown as above. At OD_600_ ~1.0, 22.5 ml of each culture was aseptically transferred to sterile 25-ml roller bottles (20 mm × 150 mm), with an aeration space of less than 10%. The caps were sealed with parafilm and the cultures were incubated at 37°C with gentle rotation (20 rpm). Samples were taken at 5 and 10 days and CFUs were counted. Oxidative stress was applied with addition of diethylenetriamine/nitric oxide-adduct (DETA/NO) to the cultures in different growth phases – OD_600_~0.6 (mid log), 1.4 (early stationary) and 2.1 (late stationary) – to a final concentration of 10 mM, and incubation continued. Samples were taken at different time points and CFU were determined. Vitamin C-mediated oxidative stress was generated as described by Vilchèze et al. (2013) with modifications. Briefly, all the three strains were grown in 7H9 medium supplemented with 10% (v/v) ADC supplement without catalase. Cells from different growth phases were drawn and exposed to Vitamin C at a final concentration of 6 mM. Samples were removed after 2 and 8 h and plated for CFU counts. For temperature stress, the strains were incubated at 37°C and 42°C, with shaking at 150 rpm. Samples were withdrawn at 24 h intervals and CFUs were recorded. For nutrient starvation, 50 ml cultures of the three strains were grown to an OD_600_ of ~0.6, harvested and washed with sterile 1X PBS (pH 6.6). The cell pellets were suspended in 50 ml sterile 1X PBS (pH-6.6) and incubated at 37°C. Viability of the cultures was monitored after 5 and 10 days by CFU counting. To study the effect of iron depletion, cells were harvested and transferred to RH defined medium (Ratledge and Hall, 1971) without FeSO_4_.7H_2_O, containing 100 μM deferoxamine mesylate salt (Sigma). Samples were plated at different time points for CFU counts. Acid stress was applied by suspending the cells grown to an OD_600_ ~0.6 in 7H9 medium with pH adjusted to 4.0. The pH was monitored at the start and end of the experiment with pH indicator strips. Samples were plated on 7H10 agar for CFU counts. Each experiment was performed in triplicate, at least thrice. Semi-quantitative RT-PCR and Live-dead staining was performed as mentioned above. The three vitamin C- treated cultures (MS8, MS9, and MS12) were lysed and plasmid stability was determined in by PCR using operon-specific (*parD2*-F and *parE2*Δ*10*-R, Supplementary Figure S3) and kanamycin specific primers (*aph*-F and *aph*-R, Supplementary Figure S3).

## Results

### Organization and Transcriptional Profile of *parDE2* TA Operon of *M. tuberculosis*

**FIGURE 1:**
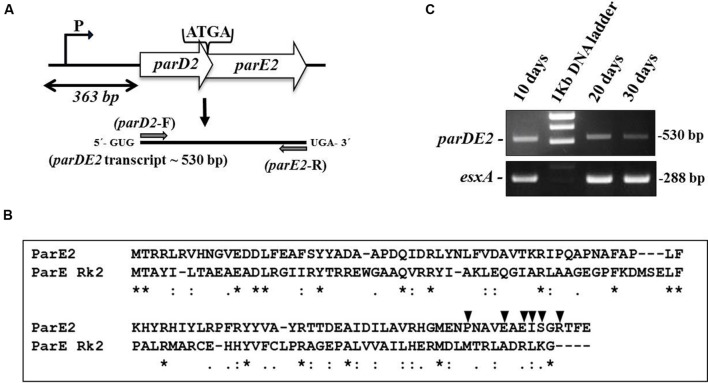
**The *parDE2* TA locus of *Mycobacterium tuberculosis*. (A)** Genetic organization of the *parDE2* operon with a 4 bp overlapping region. Gray arrows indicate where the RT-PCR primers anneal. **(B)** ClustalW alignment of MParE2 and ParE of RK2 plasmid. Identical residues (^∗^); similar residues (:). Filled triangles above MParE2 indicate the residues that were substituted by alanine. **(C)** Reverse transcriptase PCR using total RNA isolated from Mtb cells grown in broth culture for 10, 20, and 30 days.

The majority of 88 putative TA system in the *M. tuberculosis* (Mtb) genome identified by comparative genomic analysis have remained uncharacterized (Ramage et al., 2009). Since the *parDE2* TA system of Mtb was not annotated clearly, it was necessary to establish the identity of the genes and demonstrate that the ORFs are transcriptionally active and not pseudogenes. Analysis of the Mtb H37Rv genome sequence (Ramage et al., 2009) revealed the *parDE2* operon (Rv2142A-Rv2142c) as a canonical type II TA system with a 5′ antitoxin gene followed by a toxin gene, sharing a 4 bp overlap (**Figure 1A**). Blastp analysis of MParE2 and MParD2 amino acid sequences revealed only 18 and 12% sequence identity with ParE and ParD of RK2 plasmid, respectively (**Figure 1B**). The genetic organization of the operon was determined by RT-PCR using RNA prepared from different phases of growth with *parDE2* specific primers. A single 530-bp product (confirmed by DNA sequencing) corresponding to the predicted size indicated the bi-cistronic nature of the *parDE2* transcript (**Figure 1C**). No product was amplified when the RNA was used without the preceding reverse transcriptase step (data not shown). The above results revealed (i) basal level transcriptional activity of the *parDE2* locus from exponential phase till late stationary phase (**Figure 1C**) (ii) that *parDE2* is not a pseudogene and (iii) the *parD2* and *parE2* genes are transcribed as a single, bicistronic transcript.

### *parDE2* Promoter Search

**FIGURE 2:**
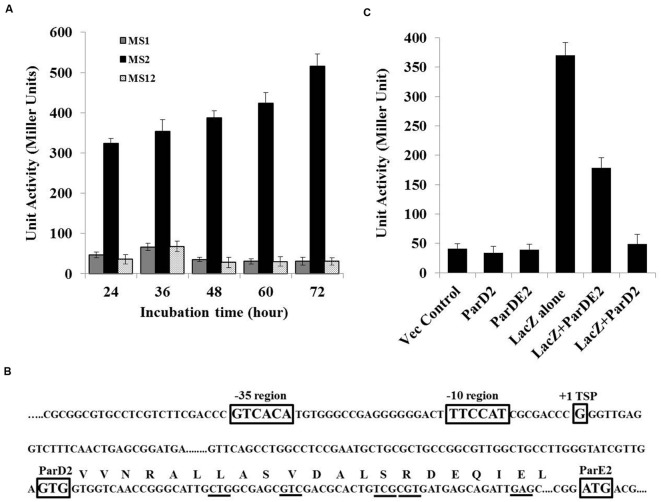
**MParDE2 Promoter identification and regulation. (A)** *M. smegmatis* recombinants viz., MS1, harboring a promoter-less *lacZ* construct; MS2, containing P*_363parD2_-lacZ* fusion construct and MS12, vector control strains were grown in 7H9 medium at 37°C and β-galactosidase activity was measured spectrophotometrically, after regular intervals. **(B)** The transcriptional start site (+1), putative -35 and -10 promoter elements along with *parD2* and *parE2* translational start sites are indicated in rectangular boxes determined by 5′-RACE PCR analysis. MParD2 amino acid residues substituted with alanine are underlined. **(C)** Regulation of P*_parDE2_* in *M. smegmatis* strains. β-galactosidase activity from different *M. smegmatis* strains harboring 363 bp *parDE2* promoter sequence fused with *lacZ* gene, co-expressing MParD2 or MParDE2 protein complex under the control of *groEL* promoter. Empty pMV261 vector, *parD2* and *ParDE2* sequences without promoter were used as negative controls. Error bars indicate mean ± SD from three independent experiments.

Since no promoter-like sequence was detected *in silico* in the *parDE2* locus by neural network prediction tools and BPROM (SoftBerry), *lacZ* fusion constructs were produced by fusing the 363 bp upstream region of the *parD2* gene (P*_363parD2_*), with the start codon of the *lacZ* reporter gene. The fusion was ligated to the *E. coli-Mycobacterium* shuttle vector, pMV261, at NotI/DraI sites and electroporated into *M. smegmatis* mc^2^ 155, producing strain MS2. The upstream 363 bp region demonstrated good promoter activity, which increased progressively with time and was highest toward post-stationary phase of the culture (**Figure 2A**). A control strain (MS1) containing promoter-less *lacZ* exhibited no significant β-galactosidase activity. These results showed that a functional promoter was located in the 363 bp upstream region of *parDE2* TA locus.

### Mapping the 5′ End of the *parDE2* Transcript and Identification of the Promoter

To define the transcriptional start point (TSP) of the P*_parDE2_* promoter, a 5′-RACE experiment was carried out. The major ~300-350 bp band produced by RACE-PCR (Supplementary Figure S1) was cloned and eight positive clones were selected and sequenced. In each case the 5′ end of the cDNA revealed an identical sequence, unambiguously indicating the transcriptional starting point.

With reference to the TSP identified above, the promoter region was defined based on the published information of nucleotide distribution within the mycobacterial promoter sequences (Newton-Foot and van Pittius, 2013). The results are summarized in **Figure 2B**. Like most of the mycobacterial promoters the *parDE*2 TSP (+1) is at a guanine (G), 103 bp upstream from the GTG start codon of the *parD2* gene and preceded at -1 by a cytosine (C). A putative –10 region centerd at 5′- TTCCAT -3′ and a corresponding -35 hexamer sequence 5′- GTCACA -3′, separated by a 19 bp optimal stretch were observed in the P*_parDE2_* promoter (**Figure 2B**). The above sequences matched well with other mycobacterial promoters recognized by σ^70^ (Newton-Foot and van Pittius, 2013). Interestingly, the identified *parDE2* promoter was identical to a -35 consensus sequence recognized by mycobacterial sigma factor J (σ^J^), induced specifically during stationary phase (Hu and Coates, 2001), oxidative stress (Hu et al., 2004) and macrophage invasion (Newton-Foot and van Pittius, 2013), suggesting its possible role in regulation of the *parDE2* locus under the above environmental conditions. Indeed, we observed regulation of the operon under oxidative stress, affecting the growth of *M. smegmatis* cells (see results below, **Figures 10A,B**). However, at this stage the regulation of *parDE2* promoter by σ^J^ under oxidative stress is purely speculative and needs to be verified in *M. tuberculosis*.

### Autoregulation of the *parDE2* TA Operon in *M. smegmatis*

**FIGURE 3:**
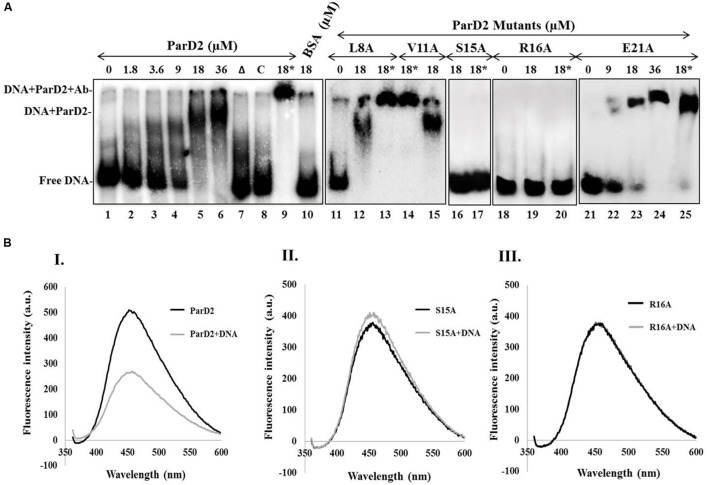
**Binding of MParD2 to promoter DNA by EMSA and ANS fluorescence assay. (A)** A 200-bp *parDE2* promoter DNA was obtained by PCR and labeled with [γ-^32^P]-ATP. The labeled DNA was incubated with different concentrations of ParD2 (lanes 1–6) or with 18 μM heat-inactivated MParD2 (lane 7) or ParD2 variant proteins. Specific and non-specific binding events are shown using unlabelled promoter DNA (lane 8) or BSA (lane 10). Lanes 9, 13, 14, 17, 20, and 25, showing super-shift EMSA using 18 μM His_6_-tagged protein and monoclonal anti-His Ab (18^∗^). Lane 11-25 shows electrophoretic mobility of the MParD2 variants. **(B)** Fluorescence emission spectra of 25 μM ANS bound to 5 μM of (I) MParD2, (II) ParD2-S15A, and (III) ParD2-R16A. The emission of ANS alone was subtracted from the observed values of each variant. Measurements were taken at 25°C with an excitation wavelength of 350 nm.

Based on the earlier reports of presence of an N-terminal ribbon-helix-helix DNA-binding domain with auto-regulatory activity in the ParD antitoxin of RK2 plasmid (Oberer et al., 2002; Dalton and Crosson, 2010), and our own observations demonstrating involvement of N-terminal residues of MParD2 in binding to the promoter DNA (**Figure 3**), we proceeded to examine the involvement of MParD2 in transcriptional regulation of the *parDE2* operon *in vivo*. For this purpose, plasmids (pMS3 and pMS4) were generated expressing *P_363_-lacZ* fusion along with MParD2 or ParDE2 complex proteins from a separate vector containing the *groEL* promoter (Methods; Supplementary Figure S2) and β-galactosidase activity was assessed in *M. smegmatis* mc^2^ 155 cells. The cells harboring P_363_-*lacZ* fusion alone (MS2) exhibited high promoter activity during log phase growth (**Figure 2C**). Presence of the two proteins in the form of ParDE2 complex repressed the promoter activity ~two-fold (**Figure 2C**). The repression was increased to ~seven-fold when MParD2 protein was present alone, suggesting that stronger repression of the promoter could be due to higher availability of free MParD2 protein in the cell (**Figure 2C**) (not limited by complex formation with MParE2), for interaction with the promoter DNA. *In vitro* interactions of MParD2 with MParE2 also supported complex formation by the two proteins (**Figure 6**). These results demonstrated that the *parDE2* promoter of Mtb is (i) transcriptionally active and (ii) negatively auto-regulated by the MParD2 protein in the surrogate *M. smegmatis* host.

### Interaction of MParD2 with the *parDE2* Promoter Region

A 200 bp DNA fragment upstream of *parD2* start codon (*P_200parD2_*) was end-labeled with [γ-^32^P]-ATP and T4 polynucleotide kinase, and used to test the DNA binding abilities of MParD2 antitoxin and its variants by EMSA. A fixed concentration of *P_200parD2_* DNA was titrated with variable amounts of MParD2 (0–36 μM, **Figure 3A**) and the optimum concentration was determined. The antitoxin MParD2 could efficiently bind to the labeled DNA fragment in a concentration-dependent manner as observed by a clear shift in electrophoretic mobility due to complex formation, with corresponding decrease in free DNA (**Figure 3A**). It was observed that 18 μM of MParD2 was sufficient to bind with the entire 20,000 cpm radiolabelled DNA probe leaving no detectable free probe and hence was used in all the assays (**Figure 3A**).

MParD2 which belongs to the *Unstab_antitox* Superfamily (*cl09921*) is predicted by PSIPRED (UCL) to contain four alpha helices. DNA binding amino acid residues in the N-terminal of MParD2 were predicted based on a Gaussian network model (Haliloglu et al., 1997) using the DNABindProt prediction tool (Haliloglu et al., 2005), and subsequently substituted by alanine. Binding of L8A, V11A and E21A, MParD2 variants to the promoter region was not affected due to mutation. However, the S15A and R16A variants showed total loss of DNA binding ability in gel-shift assays (**Figure 3A**).

DNA binding was also measured by a fluorescence competition assay employing ANS as an extrinsic fluorescent probe whose anilinonaphthalene groups fluoresce upon interaction with the surface exposed hydrophobic residues of the proteins. Addition of P*_200parD2_* DNA to the MParD2 variants-ANS complexes quenched ANS fluorescence in all except S15A and R16A variants (**Figure 3B**), which showed no or negligible quenching of the probe and reflected no DNA binding, supporting mobility shift results (**Figure 3A**). A conserved serine residue at the 15^th^ position in the ParD antitoxin of Rk2 plasmid is also shown to be involved in forming the hydrophobic core of the DNA binding motif (Oberer et al., 2002).

### Growth Characteristics of *E. coli* and *M. smegmatis* Strains Harboring *parDE2* Genes

**FIGURE 4:**
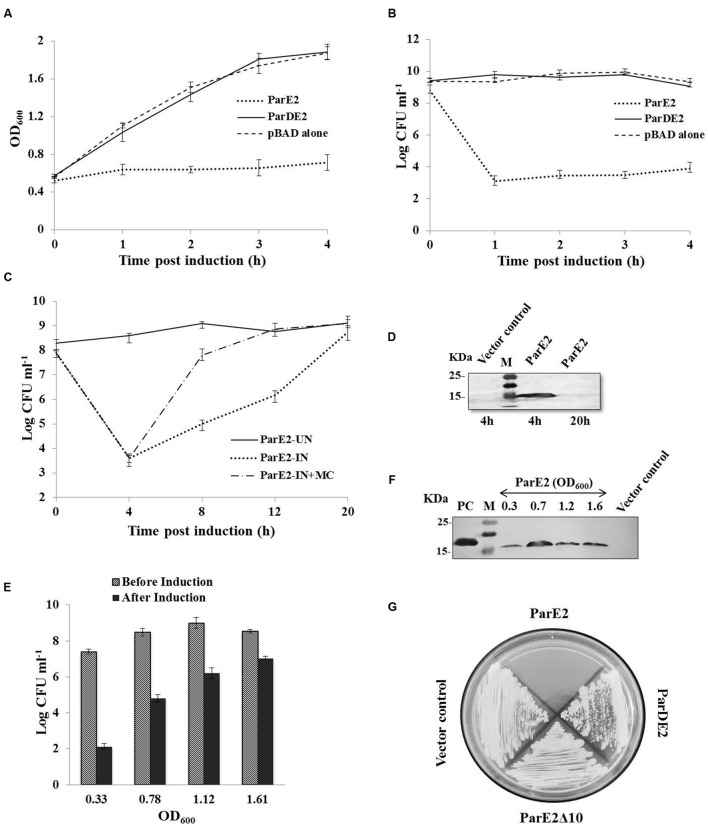
**Growth Inhibition of *Escherichia coli* and *M. smegmatis* strains expressing MParE2.** *E. coli* strains expressing *parE2* (…); *parDE2* (—); and vector control constructs (—) were grown till OD_600_ ~ 0.5, and induced with L-arabinose. **(A)** OD_600_ and **(B)** CFUs in the cultures. **(C)** MParE2 expressing EC4 cells were induced with L-arabinose and grown for 20 h (…); or after 4 h of induction cells were washed and transferred in fresh LB media containing 0.2% D-glucose (—); uninduced EC4 cells were used as control (—), and CFUs were monitored at regular intervals. **(D)** Immunoblotting of EC4 cells expressing MParE2. After induction the cell lysates (150 μg protein/well) were resolved by SDS-PAGE and the protein was detected using monoclonal anti-His probe. **(E)** ParE2 expressing EC4 cells from different growth phases were induced for 4 h and CFUs were determined. **(F)** MParE2 protein was estimated by western blotting as above. **(G)** *M. smegmatis* strains containing pMS5(*parE2*), pMS6(*parE2*Δ*10*), pMS7(*parDE2*) and pYUB1062 vector alone were streaked on 7H10 agar plates in the presence of 0.2% acetamide and 50 μg ml^-1^ hygromycin. Each time point in the growth curve is an average of three experiments with standard deviations.

The proteins encoded by *M. tuberculosis parDE2* operon were overexpressed in *E. coli* for biochemical characterization. Protein expression was induced by L-arabinose from *ara*BAD promoter in *E. coli* strains EC4 (*parE2*) and EC5 (*parDE2*). MParE2 protein was detected 2 h after induction by immunoblotting. Expression of ParE2 resulted in complete arrest of growth and replication of the cells and a drastic reduction (~5 log_10_) in CFUs vis-à-vis the *parDE2* containing strain (**Figures 4A,B**). On longer incubation (>16 h post induction), the culture slowly recovered in growth and the OD reached close to un-induced cells. When the inducer was removed and replaced with fresh medium, the recovery was faster, indicating the bacteriostatic nature of MParE2 on *E. coli*, **Figures 4C**. It is not clear if the majority of the cells die and the survivors recover or the cells are temporarily disabled and grow back when the toxin is degraded. MParE2 protein concentration in the recovered population was quite low after 20 h incubation despite the presence of plasmid in the cells (data not shown) possibly due to inducer exhaustion (**Figure 4D**).

EC4 cells were induced in different growth phases, representing early-log (OD_600_ ~0.33), late-log (OD_600_ ~0.78), early stationary (OD_600_ ~1.1) and late stationary phase (OD_600_ ~1.6). Growth of the cells was retarded in all the phases due to MParE2 toxin expression. However, the toxicity was much higher in the early growth phase resulting in ~5 log_10_ reduction in CFU in 4 h as compared to older cells from late stationary phase, in which the extent of inhibition was diminished to ~2 log_10_ only (**Figure 4E**). The magnitude of ParE2 expression was lowest in the lag phase (OD_600_~0.33), but growth inhibition was maximum (**Figure 4F**). In the exponential phase (OD_600_~0.78), relatively more protein was produced but growth inhibition was decreased. In the subsequent stages both protein expression and inhibition were lower than the exponential phase (**Figure 4F**). Since the amount of functionally active protein cannot be accurately measured here, it can be inferred that the growth phase dependent variation in MParE2 expression is not directly linked to the observed growth inhibition.

In *M. smegmatis* no recombinant colonies were obtained when pMS5 containing the toxin gene was electroporated in the tightly controlled inducible pYUB1062 system, demonstrating enhanced toxicity of MParE2 in mycobacteria. By contrast, MS6 and MS7, containing the C-terminal deletion MParE2 variant and the intact *parDE2* locus respectively, grew normally like the vector control (**Figure 4G**). Collectively, the data indicated that the transcriptionally active *parDE2* locus of *M. tuberculosis* harbors a toxin - MParE2, which is neutralized by the cognate MParD2 antitoxin.

### Effect of MParE2 Expression on Cellular Viability and Morphology

**FIGURE 5:**
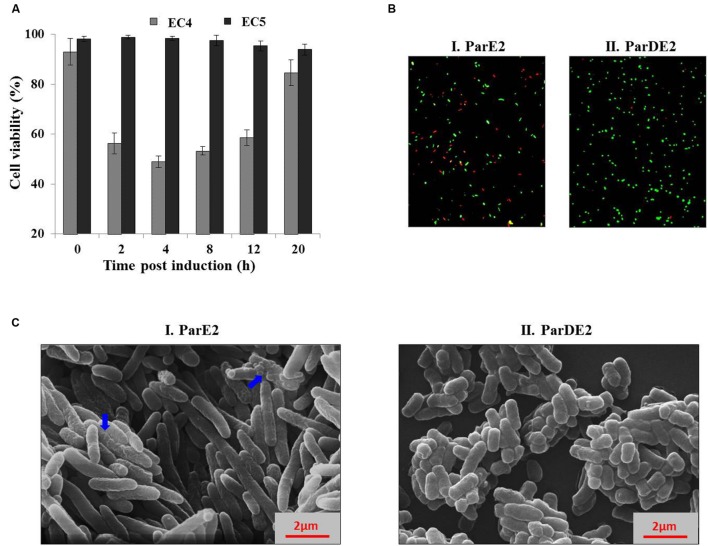
**Effect of MParE2 expression on cell viability and morphology. (A)** SYBR Green I/PI mediated cell viability assay. EC4 (ParE2) and EC5 (ParDE2) cultures were harvested at regular intervals post induction, stained and percent viability corresponding to respective G/R ratios was determined from the standard curve. The results represent average of three experiments with standard deviations. **(B)** The EC4 and EC5 cultures after 4 h induction were stained and visualized under a fluorescent microscope, showing green live cells and red dead cells. **(C)** Scanning electron microscopic view of (I) EC4 and (II), EC5 cells, 4 h post induction; blue arrows show wrinkled membranes and cellular blebbing.

To ascertain the cause of reduction in CFU counts of the MParE2 expressing cells (**Figure 4B**) during growth, the cultures were examined for viability by live-dead assay. SYBR Green I, a membrane-permeant nucleic acid stain, labels all cells in contrast to propidium iodide (PI), a membrane-impermeant stain, which enters cells with compromised membranes only. The percentage of live cells calculated in the EC4 culture were 93 ± 5.3, 56.3 ± 4.2, 49 ± 2.3, 53.3 ± 1.6, 58.6 ± 3.1 and 84.6 ± 5.2% at 0, 2, 4, 8, 12, and 20 h post-induction respectively (**Figure 5A**). In contrast, the control EC5 strain remained >90% viable till the end of the experiment (**Figure 5A**). Further, fluorescent microscopic examination of 10 fields of each culture confirmed the above results; roughly half of EC4 cells are seen to fluoresce green indicating live cells (**Figure 5B**). As expected, the number of dead cells in the MParE2 expressing culture was much higher than the MParDE2 cultures after 4 h of protein expression. Sparfloxacin-treated vector control cells displayed ~24.3 ± 3.7% viability after 4 h of exposure (data not shown). The combined results of the CFU counts and cell viability assay demonstrated that, of the ~6.3 × 10^8^ CFUs present in the culture before induction of ParE2, ~50% cells die in 4 h after induction. Thus by deduction, if the remaining 50% are alive, ~3.15 × 10^8^ colonies should be produced. However, since only ~7.7 × 10^3^ CFUs were obtained amounting to ~0.002% of the live cells, it can be assumed that a majority (99.9%) lose their ability to form colonies and enter into a viable but non-culturable state.

We further validated the live-dead assay by parallel detection of active gene expression by Reverse-transcriptase PCR (Adams et al., 2003; Trevors, 2011; Li et al., 2014). This method is based on the principle that if due to MParE2 expression, the cells become ‘viable but non-culturable’, they would retain their ability to carry out transcription, and the levels of specific mRNAs would be comparable to the control; in case the cells are dead, mRNA being unstable, the amount of transcripts would be much lower. On quantitative estimation, it was observed that the transcript levels of three reference genes (*idnT, rpoD*, and *rrsG*) were not significantly different from those of the vector control cells (Supplementary Figure S4), reiterating the point that 99.9% of the MParE2 expressing live cells were non-culturable.

To examine cellular integrity after MParE2 expression, nucleic acid release in the medium was quantified in all the strains at 260 nm. No cytoplasmic content of the bacteria was found to be released in the extracellular medium after induction (4 h) of either strain (Supplementary Figure S5). Taken together, our data revealed that MParE2 toxicity does not lead to immediate cell lysis.

MParE2 expressing *E. coli* cells exhibited an elongated phenotype as observed by SEM. The average length of the EC4 cells was 6.4 μm, ~3 times longer than the control EC5 cells with an average cell length of ~2 μm (**Figures 5C**). The latter exhibited a smooth and intact cell surface, in contrast to toxin expressing cells, that looked extensively corrugated, rough and displayed an uneven surface with dimples.

### *In Vitro* Interaction of MParD2 Antitoxin with MParE2 Toxin

**FIGURE 6:**
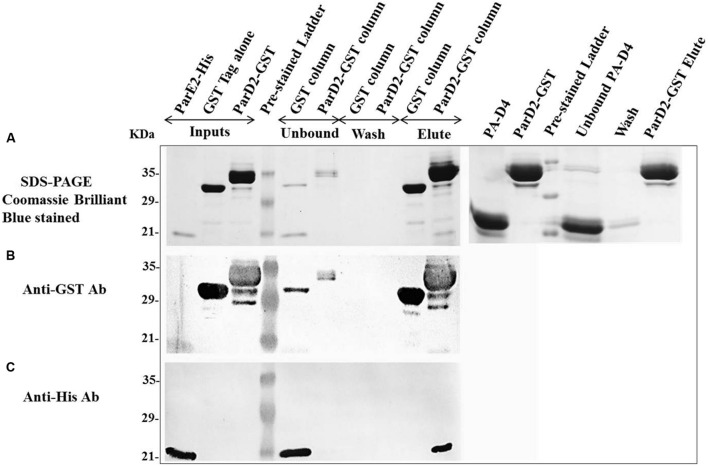
**Interaction of MParD2 with MParE2 by GST Pull-down assay.** The partially purified His6X-MParE2 protein mixture was incubated with GST-MParD2 or GST tag alone, immobilized on glutathione-beads. MParE2 bound to the beads was detected by **(A)**, SDS-PAGE followed by coomassie blue staining. His6X-Domain 4 of Protective Antigen of *Bacillus anthracis* was used as non-specific protein-prey control. **(B)** Immunoblotted with Anti-GST Antibody. **(C)** Anti-His Antibody. Pre-stained low range ladder (Bio-Rad) was used in all the experiments.

After neutralization of MParE2 toxicity by the MParD2 protein during *in vivo* growth, *in vitro* interaction of the two proteins was examined. SDS-PAGE analysis revealed the presence of both His-MParE2 and GST-MParD2 proteins in the same fraction eluted from the glutathione-sepharose affinity column. This was confirmed by immuno-blotting using anti-GST and anti-His monoclonal antibodies (**Figure 6**). In the absence of GST-MParD2, His-MParE2 did not bind to the GST column and eluted in the unbound fraction. Also His-tagged PA-D4 did not bind to GST-MParD2 on column and most of it came out in the unbound fraction, demonstrating specificity of MParD2-MParE2 interaction (**Figure 6**).

### Inhibition of Mtb DNA Gyrase Supercoiling Activity by MParE2 Toxin

Reconstitution of the DNA gyrase holoenzyme (A_2_B_2_) from equal amounts of GyrA and GyrB subunits and further purification with gel filtration chromatography provided the active enzyme corresponding to an apparent mass of 345 kDa, confirmed by blue-native PAGE analysis (data not shown). The specific activity of the purified, recombinant Mtb DNA gyrase holoenzyme was approximately 2.3 × 10^3^ U mg^-1^; much lower than that of *E. coli* and *Bacillus subtilis* (>10,000 U mg^-1^; Orr and Staudenbauer, 1982; Reece and Maxwell, 1989). No supercoiling was observed when ATP was omitted from the reaction mixture (data not shown).

**FIGURE 7:**
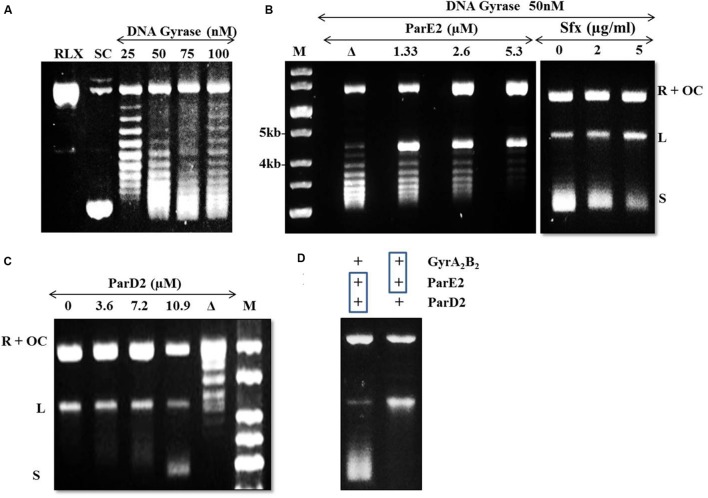
**Modulation of DNA gyrase activity of Mtb by MParE2 and MParD2 proteins.** pBR322 plasmid DNA (1 μg) was used as a substrate in the DNA gyrase supercoiling assay. **(A)** DNA gyrase supercoiling assay. Relaxed (RLX), negative control; supercoiled (SC), positive control; L, linearized and OC, open circular form of pBR322. **(B)** inhibition of DNA gyrase activity by MParE2. All the samples contained 1 unit of DNA gyrase holoenzyme. M, 1 kb DNA ladder; Δ, heat denatured 2.6 μM MParE2. For control experiments 0, 2 and 5 μg ml^-1^ sparfloxacin was used. **(C)** Inactivation of MParE2 toxicity by MParD2. 5.3 μM MParE2 pre-mixed with 0, 3.6, 7.2, and 10.9 μM MParD2 prior to mixing with DNA Gyrase. Δ, heat denatured 10.9 μM MParD2. **(D)** Left lane, 5.3 μM MParE2 and 10.9 μM MParD2 pre-incubated for 30 min and added to 1 unit of DNA gyrase reaction mix; right lane, 5.3 μM MParE2 pre-incubated with 1 unit of DNA gyrase for 30 min and 10.9 μM MParD2 added to assay mix.

The DNA gyrase poisons such as ParE, CcdB and quinolone antibiotics stabilize/trap the reaction intermediates by binding to the cleaved DNA-gyrase complex and stall replication fork movement (Bernard and Couturier, 1992; Jiang et al., 2002). In the agarose gel, accumulation of linear dsDNA species after addition of SDS and proteinase K indicates inhibition of gyrase activity. Based on this mechanism, we examined the mode of action of the mycobacterial MParE2 on DNA gyrase. The DNA supercoiling assay using a relaxed pBR322 plasmid as substrate is shown in **Figure 7A**. MParE2 inhibited DNA gyrase activity, resulting in accumulation of the cleaved DNA intermediate, while the amount of supercoiled DNA decreased progressively with increasing toxin concentration (**Figure 7B**). When MParE2 was heat inactivated, the supercoiling reaction progressed normally, converting most of the relaxed pBR322 DNA into the supercoiled form while the concentration of intermediate cleavage product reduced drastically (**Figure 7B**), indicating that the inhibition was indeed due to MParE2 protein. In control experiments, addition of 2 and 5 μg ml^-1^ sparfloxacin to the assay mixture, also resulted in accumulation of cleavage intermediates and gradual decrease in the amounts of supercoiled DNA as seen with MParE2 (**Figure 7B**).

### Reversal of DNA Gyrase Inhibition by MParD2

The inhibitory effect of MParE2 on Mtb DNA gyrase mediated supercoiling was blocked by pre-incubation of the toxin with MParD2 protein for 30 min (**Figure 7C**). Approximately 10.9 μM MParD2 antitoxin (~2 times) was sufficient to abrogate the inhibitory effect of 5.3 μM MParE2 toxin in the *in vitro* assay. The same amount of heat inactivated MParD2 was unable to neutralize the inhibition caused by MParE2 (**Figure 7C**); only a few intermediates of the trapped cleavable complexes were observed. Interaction of MParD2 with MParE2 for 30 min before adding to the rest of the reaction mixture allowed the gyrase reaction to proceed normally (**Figure 7D**). However, MParD2 could not reverse the inhibition when DNA gyrase was pre-incubated with MParE2, indicating that the antidote is unable to rescue an ‘MParE2-poisoned’ DNA-gyrase complex (**Figure 7D**) unlike other known antitoxins (Pedersen et al., 2003). The *in vitro* activity of MParD2 is consistent with its *in vivo* role in preventing ParE2 mediated killing.

### Interaction of MParE2 with Mtb Gyrase A and B Subunits

**FIGURE 8:**
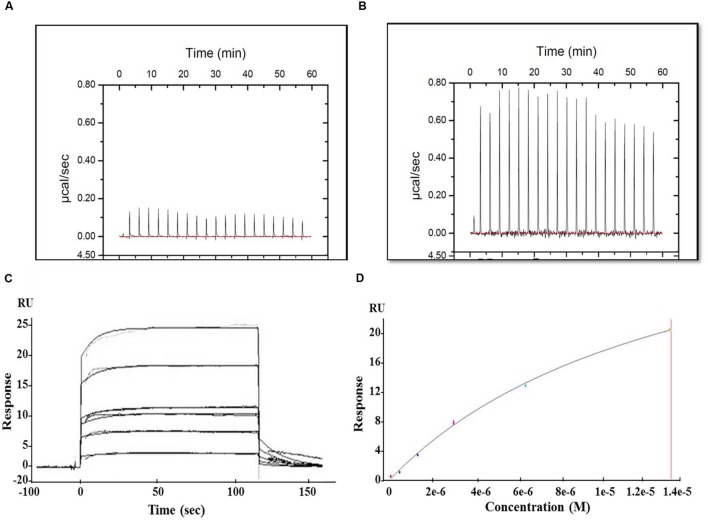
**Interaction of MParE2 with GyrB by SPR and ITC studies.** ITC measurements were performed at 25°C using the MicroCal iTC200 system (GE Healthcare). Binding isotherms were integrated and analyzed using Origin v7.0 software (MicroCal). **(A)** 70 μM of GyrA and **(B)** 70 μM of GyrB were titrated against 700 μM ParE2. SPR analysis using BIAcore T200. His_6_-GyrB subunit was captured by Anti-His MAb immobilized on a CM4 sensor chip at a surface density of 2,500 RU. The analytes were injected over the immobilized ligands and analysis was carried out with different dilutions at a flow rate of 20 μl min^-1^ followed by a dissociation time of 600 s. **(C)** Sensogram (Resonance/Responsive units vs. Time) shows the binding kinetics of MParE2 with GyrB at different dilutions. **(D)** Best fit curve of change in RUs with varying MParE2 concentrations.

The interaction between MParE2 and the individual gyrase subunits were examined by ITC and SPR. In ITC, the thermal profile of Mtb GyrB and MParE2 displayed positive peaks indicating an endothermic binding interaction (**Figure 8B**). However, the binding was weak, as indicated by lack of saturation. No interaction of MParE2 was detected with the Mtb GyrA subunit (**Figure 8A**). Consistent with ITC data, the association of MParE2 toxin with GyrB subunit, when monitored by SPR, indicated that the interaction was moderate in strength with a K_d_ M of 4.75E-6 (**Figures 8C,D**). No binding was observed with GyrA under these conditions. In a control experiment, interaction of sparfloxacin with GyrA showed K_d_ *M* = 9.228E-8 (data not shown) indicating that the GyrA bound to the chip was properly folded and immobilized. From these results it is concluded that MParE2 interacts with GyrB subunit albeit with relatively lower affinity under *in vitro* conditions. The ATPase activity of GyrB in the presence of varying concentrations of MParE2 toxin was measured using radiolabelled γ-^32^P-ATP as substrate. No effect on the ATPase activity was observed after 1 h of incubation over a range of 0.66 to 16 μM of MParE2, demonstrating that it does not interfere with the ATP binding site of GyrB (Supplementary Figure S6).

### Identification of C-terminal Amino Acid Residues Critical for Toxicity of MParE2 in *E. coli*

**FIGURE 9:**
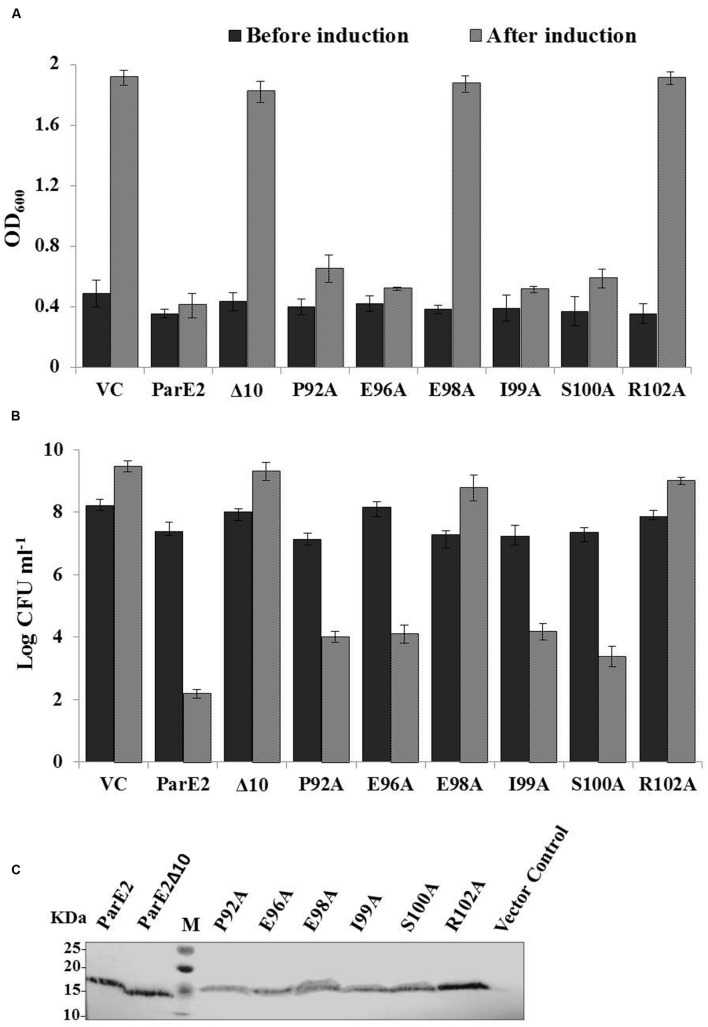
**Effect of C-terminal mutations on toxicity of *parE2*.** *E. coli* strains expressing MParE2 C-terminal deletion (10 residues) and six point mutants, P92A, E96A, E98A, I99A, S100A, and R102A, were grown in LB broth and induced with L-arabinose and growth was measured. **(A)** Optical density; **(B)** viable counts of the cultures represent average of three independent experiments with standard deviations. **(C)** Immunoblotting of MParE2 variants. Equal amount of total protein extract (150 μg/well) of the strains were subjected to immunoblotting using Anti-His monoclonal antibody. Wild type His6X-MParE2 was used as a positive control.

The residues essential for biological activity of MParE2 were identified by deletion and site-directed mutagenesis. *In silico* structural analysis of MParE2 revealed that a 23-residue stretch at the C-terminal of the protein is free, forming no secondary structure. Hence, we assumed that mutations in this region are not likely to destabilize the protein. To this end, we created MParE2 deletion variant, by deleting 10 amino acid residues from the C-terminal end. It was found that deletion of only 10 residues from the C-terminus rendered it non-toxic both in *E. coli* and *M. smegmatis* (**Figure 4F**). The *E. coli* cells expressing wild type MparE2 revealed ~5 log_10_ decrease after 2 h of induction compared to MParE2Δ10 strain (EC6), which grew normally, showing ~1 log_10_ increase in the CFU (**Figures 9A,B**).

Following the lead from C-terminal deletion, we proceeded to identify the individual residues responsible for toxic activity; six amino acid residues namely: P92, E96, E98 I99, S100, and R102, located in the C-terminus were altered to alanine by SDM and their effect on cellular growth was monitored. The E98A and R102A variants showed total loss in toxicity as the cells grew normally after induction (**Figure 9A**). The effect of alanine substitutions at positions P92A, E96A, I99A, and S100A reduced MParE2 toxicity to varying degrees but was not as dramatic as E98A and R102A (**Figure 9B**). Protein expression in each case was confirmed by immuno-blotting after 4 h induction (**Figure 9C**). Thus identification of the specific C-terminal residues critical for toxicity of the protein is an important step in understanding MParE2 mediated intoxication of cells.

### Stress Response of *M. smegmatis* Containing the *parDE2* Operon

Based on the previously described results demonstrating activity and autoregulation of the M*parDE2* promoter in *M. smegmatis* (**Figure 6**), we designed the following experiment on the premise that if, in response to specific environmental stresses, the M*parDE2* operon and certain proteases are activated, the cells will produce free MParE2 protein owing to the differential stability of MParD2. The toxic effect would be visible on cellular viability and quantifiable by CFU counts. Hence *M. smegmatis* MS8 containing the wt-*parDE2* operon, MS9 containing C-terminal truncated *parDE2* operon and MS12 vector control strains were generated and subjected to a variety of stress conditions encountered within the host.

**FIGURE 10:**
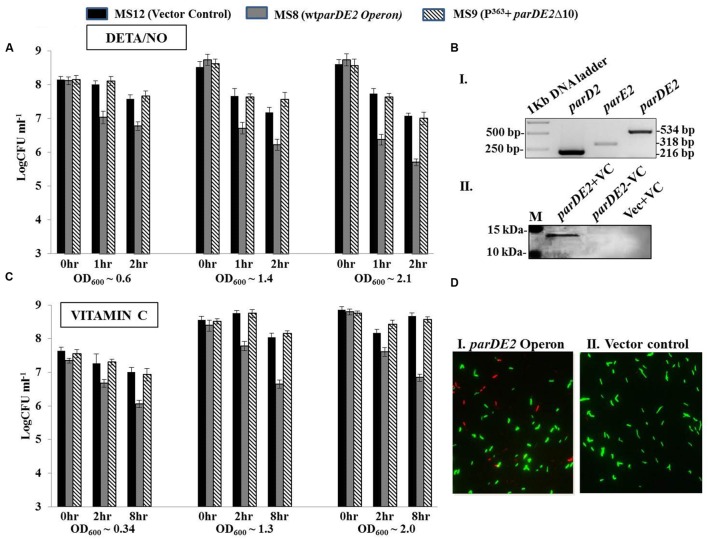
**Modulation of *MparDE2* locus in *M. smegmatis* under stress conditions.** Cultures from different ages were subjected to different stress conditions. CFU counts were determined before and after stress treatment. **(A)** Cultures from different growth phases, OD_600_ ~0.6 (mid log), 1.4 (early stationary) and 2.1 (late stationary) were exposed to oxidative stress with 10 mM DETA/NO, and CFUs were determined after 1 and 2 h. **(B)** Cells in different growth phases were exposed to 6 mM Vitamin C. Samples were removed after 2 and 8 h, washed and plated for CFU counts. The data presented are mean ± SD of triplicate samples and are representative of three individual experiments. **(C)**, (i) Semi-quantitative RT-PCR analysis of *parD2*, *parE2*, and *parDE2* transcripts using total RNA from MS8 cells; (ii) immunoblotting of Vitamin C-treated (8 h) MS8 cell lysate, using ParE2 specific polyclonal antisera raised in Balb/c mice. M, pre-stained protein ladder (PageRuler). **(D)** Live-dead staining of *M. smegmatis* recombinants after 8 h of Vitamin C treatment.

Diethylenetriamine/nitric oxide-adduct, used as a reagent to produce oxidative stress in cell culture experiments, caused uniform reduction in the CFUs with time in all the three *M. smegmatis* strains. Hence to minimize its effect the oxidative stress was evaluated in younger cells (OD_600_ ~0.6) with short exposure time. Early log phase cultures of MS8, after 1–2 h of DETA/NO exposure, exhibited ~0.7–1 log_10_ decrease in viability compared to MS9 strain and MS12 strains. The effect of DETA/NO was highly pronounced in the late log and stationary phases with ~1.5 log_10_ decrease in CFUs as compared to the two control strains (**Figure 10A**). However, this decrease could also be due to reduction in fitness of the population with time, exacerbating the effect of MParE2.

The three strains were also exposed to vitamin C (VC), which is known to produce oxidative stress. Unlike DETA/NO, no toxicity was observed on the growth rate or cell viability in the control strains with 6 mM VC during the course of 8 h, while MS8 cells showed ~1.4–1.5 log_10_ reduction in viability (CFU) in all the growth stages (**Figure 10B**). The observed reduction in CFU under oxidative stress could be due to two reasons; either the cells are dead or they have become viable but non-colony forming like *E. coli*. Molecular analysis of the VC-treated MS8 strain by semi-quantitative RT-PCR and western blotting indicated transcription of both the *parD2* and *parE2* genes (**Figure 10C**), and presence of MParE2 protein in the cells (**Figure 10C**). Visual examination of the Live-dead stained cultures, a widely accepted method to measure cellular viability (Frampton et al., 2012), showed only marginal increase in the number of dead cells in VC treated MS8 than in the MS12 culture (**Figure 10D**), which did not match with the CFU counts. Lastly, presence of the plasmid in all the three strains was confirmed by PCR amplification and the possibility of plasmid instability was ruled out (Supplementary Figure S7A). Thus, MParE2 seems to generate VBNCs in *M. smegmatis*, as observed in *E. coli* before.

Colony forming units enumeration of MS8 cells subjected to hypoxia revealed differential regulation in comparison to MS9 and MS12 strains. A general decrease in CFUs of all the strains with time was observed during the experiments presumably due to natural cell death. After exposure to hypoxia a decrease in CFU of ~0.5–0.6 log_10_ occurred in wt-*parDE2* harboring MS8 cells at 5 and 10 days, respectively (Supplementary Figure S7B), suggesting that the *parDE2* operon was regulated under hypoxia. No significant change in cell density or CFUs of the *M. smegmatis* strains was observed under nutrient starvation and heat stress, suggesting that the *parDE2* operon did not respond to these stress conditions (Supplementary Figures S7E,F). Similarly, the operon remained unaffected under iron depletion or acid stress (Supplementary Figures S7C,D).

## Discussion

To cope with environmental stresses within the host, *M. tuberculosis* enters a transient state of dormancy characterized by a decrease in growth rate and metabolic activity. Since these cells do not multiply, they escape detection by culture-based diagnostic methods. This small, antibiotic-tolerant population, called the persister is an impediment in eradication of tuberculosis. In this light, MParE2 protein, an inhibitor of DNA gyrase, a key enzyme involved in the process of DNA replication appeared to be an ideal candidate to explore.

The proteins encoded by Mtb *parDE2* operon were overexpressed in *E. coli* for biochemical characterization and their biological activity was examined in the saprophytic strain *M. smegmatis*, as it is physiologically closer to *M. tuberculosis* H37Rv but lacks homologs of most of the predicted TA loci of Mtb (Ramage et al., 2009). This provided an ideal system to investigate the *parDE2* genes in-isolation, as redundancy, cross-talk and functional overlap commonly observed between TA systems hamper their biochemical characterization (Zhu et al., 2010; Sala et al., 2014). Overall, the growth inhibition of both *M. smegmatis* and *E. coli* by MParE2, and its reversal by the cognate MParD2, indicated that MParE2 toxicity is not restricted to the mycobacterial genus only, given the conservation of DNA gyrase among bacteria. Co-transcription of the *parDE2* operon as a single primary transcript, and neutralization of MParE2 toxicity by MParD2 both *in vivo* and *in vitro*, validated MParDE2 as an archetypal TA system of Mtb.

MParE2 differs from other toxins like Rk2-ParE (Jiang et al., 2002) and CcdB (Bernard and Couturier, 1992) in binding to the GyrB and not the GyrA subunit. The DNA gyrase poisons either bind to and stabilize the gyrase-DNA cleavage complex, a hallmark of stalled DNA gyrase activity or inhibit the ATPase activity encoded in the GyrB subunit (Collin et al., 2011). Since the ATPase activity encoded in the N-terminus of GyrB, was not affected in our studies, we surmise that the binding residues are far from the active site, implying involvement of the C-terminal domain in MParE2 binding. The binding sites on the target DNA gyrase holoenzyme vary for different inhibitors, as shown by susceptibility of a CcdB resistant *E. coli* to ParE toxin (Roberts et al., 1994). The amino acid residues involved in binding of CcdB, quinolones and some ParE proteins all map in the GyrA subunit (Madurga et al., 2008), mostly within the QRDR (quinolone resistance-determining region), while coumarins and cyclothialidines are known to bind to the GyrB subunit (Madurga et al., 2008; Collin et al., 2011). Thus, though MParE2 inhibits gyrase activity by stabilizing the cleavage complex like other TA toxins, its binding preference is for GyrB and not GyrA like the others. From the available data it is not possible to predict how MParE2 might be inhibiting the enzyme activity. It could bind to GyrB subunit and cause a conformational change affecting catalytic activity of the holoenzyme. Alternatively, binding of MParE2 may stabilize the gyrase-DNA complex in a cleaved state by bringing the residues of the quinolone binding pocket closer within the QRDR, as shown by Heddle and Maxwell (2002). Solving the structure of the Mtb DNA-gyrase-MParE2 complex will shed more light on the mechanism of inhibition of gyrase by MParE2.

The rapid fall in CFUs (~10^5^ fold) of *E. coli* upon MParE2 expression, initially thought to be cell death due to toxicity, did not match with live cell counts (more than 50%) obtained by live-dead staining assay. Quantitative estimation of transcriptional activity of specific genes, live-dead staining and CFU counts together demonstrated that majority of the live cells were viable but non-culturable (VBNC), a survival strategy adopted by many bacterial species (Li et al., 2014; Ayrapetyan et al., 2015). Thus upon MParE2 expression, *E. coli* responded in three different ways, (i) half the population died, presumably an altruistic response for self-preservation of the population till the return of favorable conditions (Lennon and Jones, 2011; Pinto et al., 2011), (ii) the live cell population stochastically differentiated into two phenotypically distinct subpopulations wherein a major portion lost their ability to form colonies and entered a quiescent state (Ayrapetyan et al., 2015), and (iii) a minor (~0.002%) population of the live cells retained their ability to form colonies, showed slow growth rate as the only sign of intoxication and are believed to be progenitors of persister cells (**Figure 4B**). The reason for the observed phenotypic diversity of the population has been attributed to variation in the level of toxin expression in the cells, suggestive of a bistability/tristability-like phenomenon (Dubnau and Losick, 2006; Feng et al., 2014a). A similar phenomenon was reported earlier (Shah et al., 2006) in a recombinant *E. coli* culture expressing green fluorescent protein under a ribosomal promoter, active in dividing cells only. A small number of cells that stochastically entered into dormancy appeared dim due to diminished cellular activity. Thus, we hypothesize that though MParE2 expression caused growth inhibition of the entire population (**Figures 4AB**), the magnitude of toxin protein expression may be variable in individual cells, determining their fate. We assume that majority of the viable cells that crossed a threshold showed the VBNC phenotype, while those below the threshold were less affected and do not show the extreme effects of toxin. This is similar to the toxin threshold-dependent generation of VBNC and persisters, reported earlier in case of HipBA TA module (Rotem et al., 2010).

In the light of involvement of TA systems in stress alleviation/tolerance (Lewis, 2007; Wu et al., 2012), activation of the *parDE2* operon and release of free toxin specifically under oxidative stress, a condition known to prevail in the host macrophages, strongly argues for its relevance in host niches. As observed in *E. coli*, expression of MParE2 in *M. smegmatis* also stochastically generated a minor colony forming and a major non-culturable quiescent population, suggesting operation of a common bet-hedging strategy mediated by the toxin in the two evolutionarily distant bacterial species. Our results together suggest that ParDE2 TA system of Mtb could be a key component of the mycobacterial stress tolerance arsenal to evade the host generated immune onslaught.

## Conclusion

Considering the involvement of TA modules in regulating critical cellular functions like growth and development, programmed cell death and virulence determinants, e.g., biofilm formation and persistence of pathogenic bacteria (Wen et al., 2014), investigation of the *parDE2* operon of Mtb looks promising for developing strategies to control the disease. Differentiation of MParE2 expressing culture into phenotypically distinct VBNC populations, points to an ingenious program for long-term survival and producing a chronic infection. The inherent behavioral heterogeneity revealed by MParE2 expressing cells suggests an effective mechanism of adaptation to environmental stresses potentially leading to persistence and merits further investigation. Work is in progress to study the role of *parDE2* genes in the pathogenicity of Mtb.

## Author Contributions

MG: Planned the experiments, performed experiments, analyzed data, wrote and revised the manuscript. NN: Performed experiments, analyzed data, wrote the paper. MC: Performed experiments, analyzed data. RS: Planned the experiments, analyzed data, revised the manuscript. RB: Planned the experiments, analyzed data. NB: Planned experiments; analyzed data; wrote and revised the manuscript.

## Conflict of Interest Statement

The authors declare that the research was conducted in the absence of any commercial or financial relationships that could be construed as a potential conflict of interest.

## Footnotes

1 http://www.who.int/mediacentre/factsheets/fs104/en/
