# Supplementary material for: The Chromosomal parDE2 Toxin–Antitoxin System of Mycobacterium tuberculosis H37Rv: Genetic and Functional Characterization
Source: Front Microbiol. 2016 Jun 14;7:886. doi: 10.3389/fmicb.2016.00886 (PMC4906023; doi:10.3389/fmicb.2016.00886)
Supplement: Supplementary file 1 [file Presentation_1.PDF]

Supplementary Figures and Legends

Fig. S1 5´-RACE PCR analysis

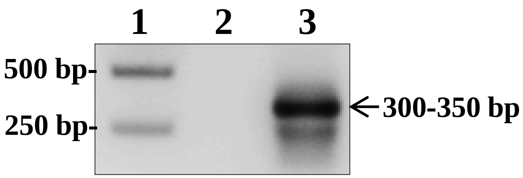

Agarose gel electrophoresis of the **5´-**RACE-PCR amplification. Lanes: 1, 1-kb Plus DNA Ladder (Fermentas); 2, negative control (no RT); 3, RT-PCR using *parDE2* transcript.

Fig. S2 Vector map of pMS2 harbouring *parD2* or *parDE2*

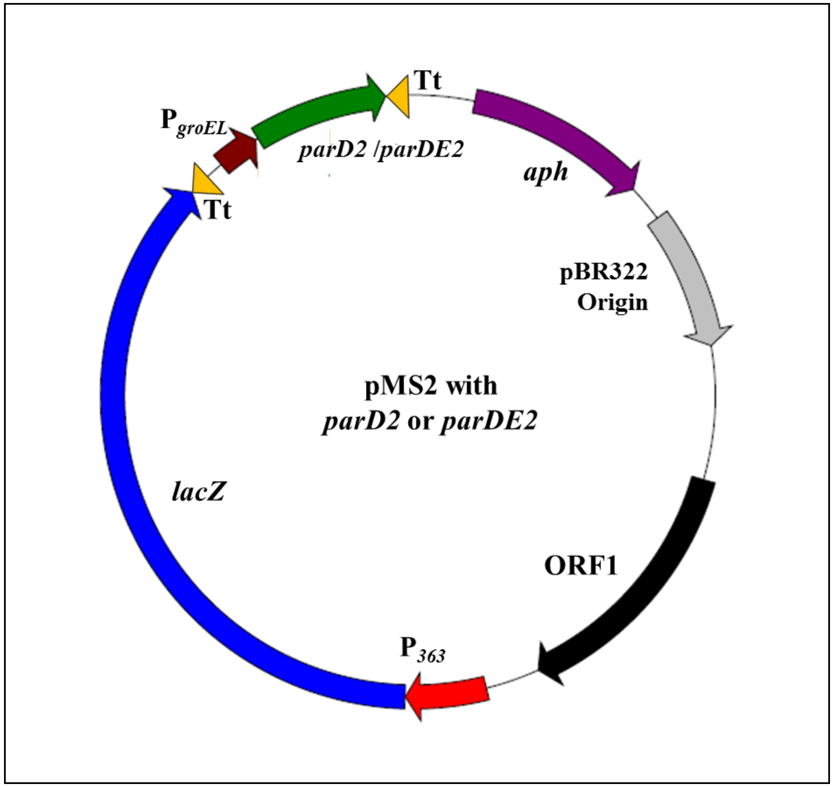

Vector map of pMS2 harboring the *lacZ* gene with its regulatory sequences, under the control of $P_{363parD2}$, using NotI and DraI sites. The *parD2* or *parDE2* coding sequences were placed downstream of the containing *groEL* promoter of pMS2, at restriction sites EcoR1 and HindIII, to form pMS3 or pMS4 respectively .

Fig. S3 Primers used in this study

Primers for *parE2*-wild type

parD2-F : 5′ – CATATGGTGGTGGTCAACCGGGC – 3′

parD2-R : 5′ – CTCGAGTCATCCGAGCCGGGCGC – 3′

parE2-F : 5′ – CTCGAGATGACGCGCAGGCTGCGCGT – 3′

parE2-R : 5′ – AAGCTTTCACTCGAAGGTGCGGCC – 3′

esxA-F : 5′ – ATGACAGAGCAGCAGTGG – 3′

esxA-R : 5′ – CTATGCGAACATCCCAGT – 3′

aph-F : 5′ – ATGAGCCATATTCAACGGGA – 3′

aph-R : 5′ – TTAGAAAAACTCATCGAGCA – 3′

Primers for C-terminal deletion and point mutants (reverse primers)

*parE2*Δ10-R : 5′ – AAGCTTTCAGACCGCGTTCGGGTT – 3′

parE2/R102A : 5′ – ATCTCTGGCGCCACCTTCGAGTGA – 3′

parE2/S100A : 5′ – GAGATCGCTGGCCGCACCTTCGAGTGA – 3′

parE2/I99A : 5′ – GCTGAGGCCTCTGGCCGCACCTTCGAGTGA – 3′

parE2/E98A : 5′ – GAGGCTGCGATCTCTGGCCGCACCTTCGAGTGA – 3′

parE2/E96A : 5′ – GCGGTCGCGGCTGAGATCTCTGGCCGCACCTTCGAGTGA – 3′

parE2/P92A : 5′ – GAGAACGCGAACGCGGTCGAGGCTGAGATCTCTGGCCGCACCTTCGAGTGA – 3′

Primers for *parD2* point mutants

parD2/L8A/F : 5′ – CAGATCCATATGGTGGTGGTCAACCGGGCATTGGCGGCGAGCGTCG – 3′

parD2/V11A/F : 5′ – CAGATCCATATGGTGGTGGTCAACCGGGCATTGCTGGCGAGCGCCGACGCACTGT – 3′

parD2/S15A/F : 5′ – CAGATCCATATGGTGGTGGTCAACCGGGCATTGCTGGCGAGCGTCGACGCACTGGCGCGTGATGAGC – 3′

parD2/R16A/F : 5′ – CAGATCCATATGGTGGTGGTCAACCGGGCATTGCTGGCGAGCGTCGACGCACTGTCGGCTGATGAGCAGA – 3′

parD2/E21A/F : 5′ –CAGTACCATATGGTGGTGGTCAACCGGGCATTGCTGGCGAGCGTCGACGCACTGTCGCGTGATGAGCAGATTGCGCTCGTCGAGC – 3′

parD2-R : 5′ – CAGATCCTCGAGTCCGAGCCGGGCGCGGATCCGCTTGTCGAAGTCATCAATGGTGGACCA – 3′

Oligos used to generate wild type MParE2 and MParD2, MParE2 deletion (ParE2Δ10) and point mutants (P92A, E96A, E98A, I99A, S100A and R102A), and MParD2 point mutants (L8A, V11A, S15A, R16A and E21A). ESAT-6 (*esxA*) and Kanamycin resistance gene (*aph* from Tn903) specific primers have also been mentioned.

Fig. S4   qPCR analysis of MParE2 expressing cells

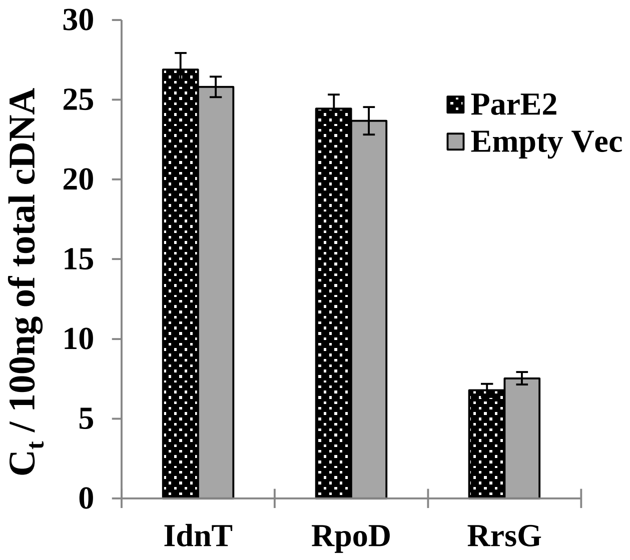

The cycle threshold levels/100 ng of total cDNA was almost equal for all the three reference genes (*idnT*, *rpoD* and *rrsG*) in MParE2 expressing EC4 cells, as compared to the vector control cells.

Fig. S5 Nucleic acid release

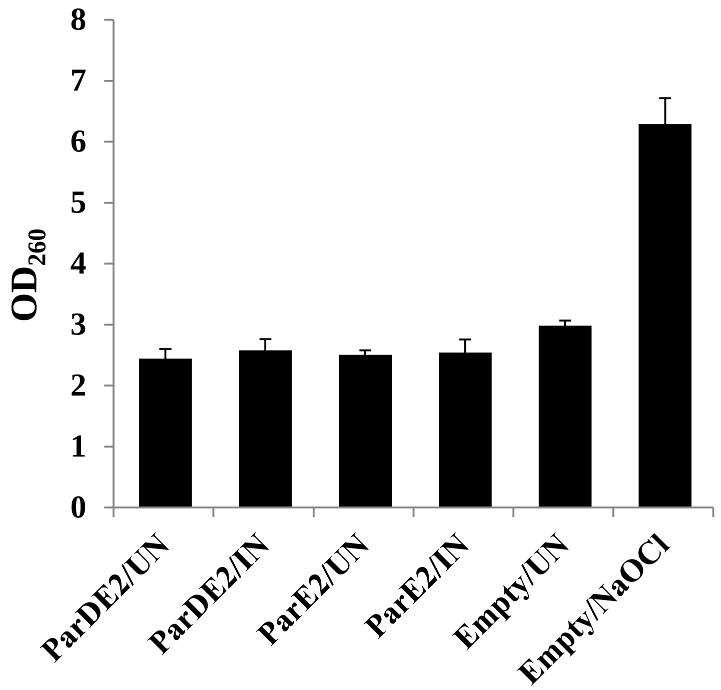

EC4 (ParE2) and EC5 (ParDE2) containing strains were induced with L-arabinose and the presence of the 260 nm absorbing materials in extracellular medium was evaluated after 4h. Sodium hypochlorite (NaOCl) treated *E. coli* TOP10 containing pBAD/HisA was taken as control. The experiment was done in triplicate for each condition, and the data was normalized against the absorbance of 1X PBS at the same wavelength (UN- untreated cells, IN- arabinose-induced cells).

Fig. S6 ATPase assay

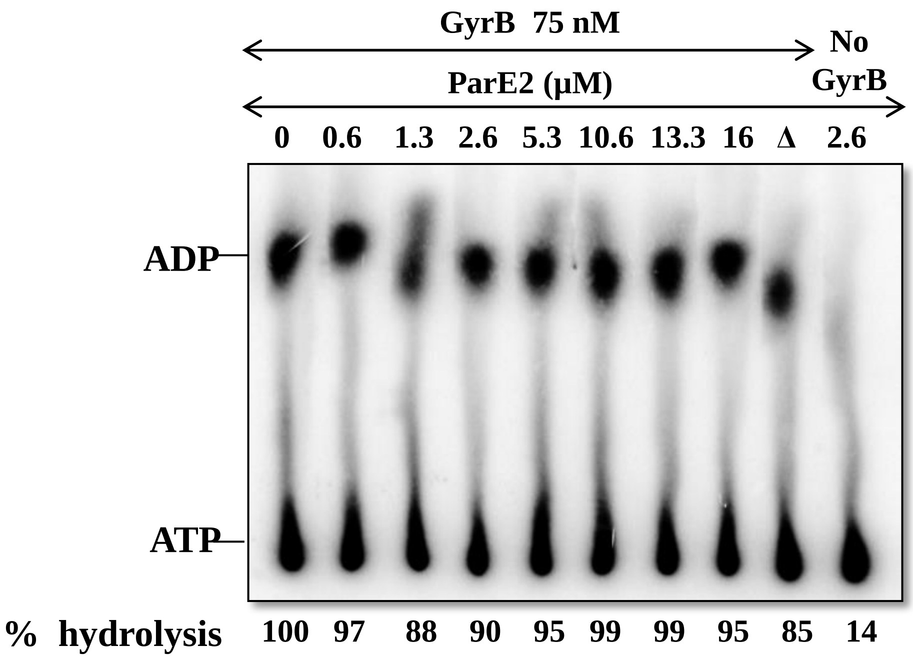

For all the ATPase assays 75nM GyrB was titrated against 0 to 16 µM MParE2 keeping other parameters constant. In each case the signal intensities of the ATP hydrolysis were analyzed by NIH Image J software.

Fig. S7 Regulation of M*parDE2* locus in *M. smegmatis* under stress conditions

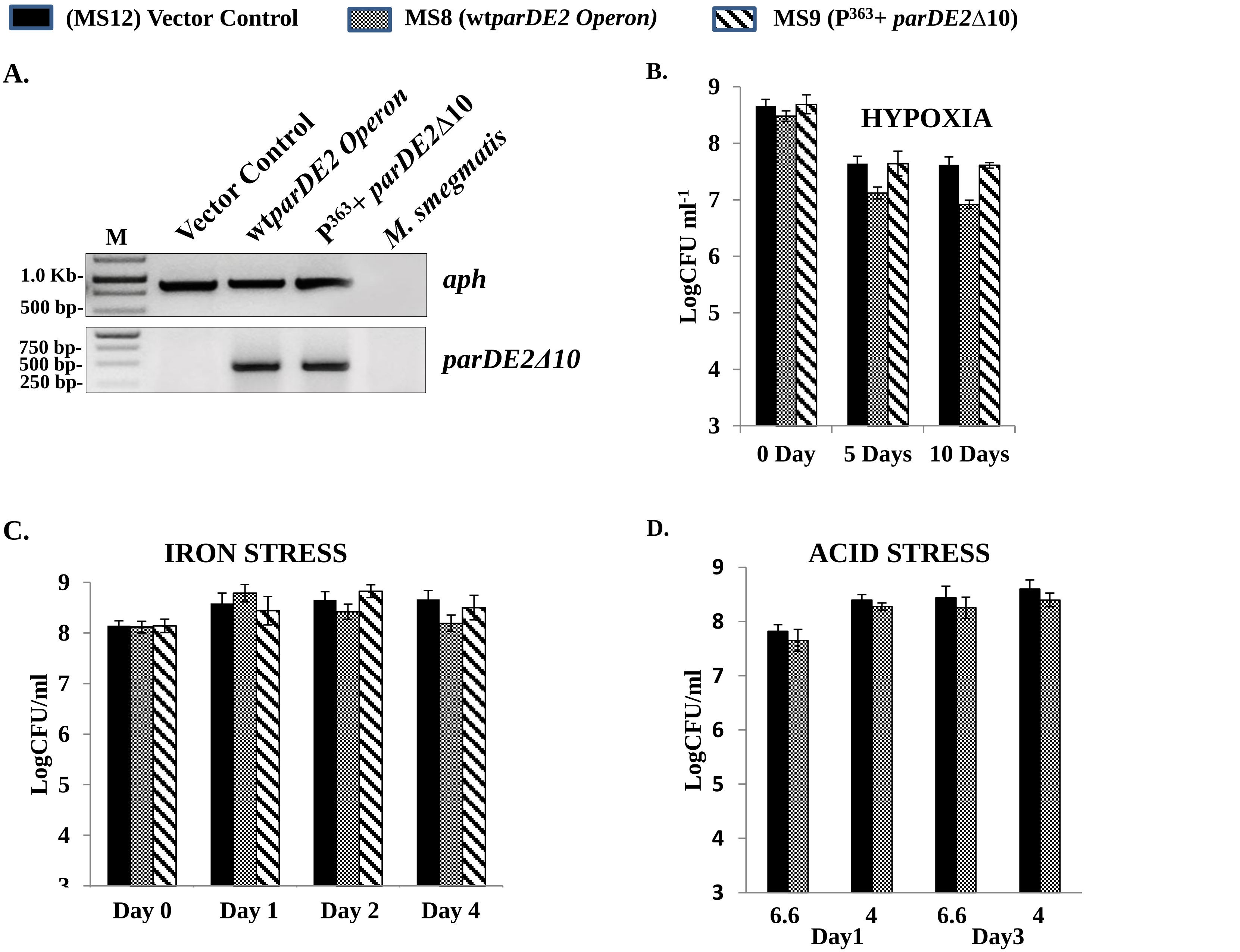

E.

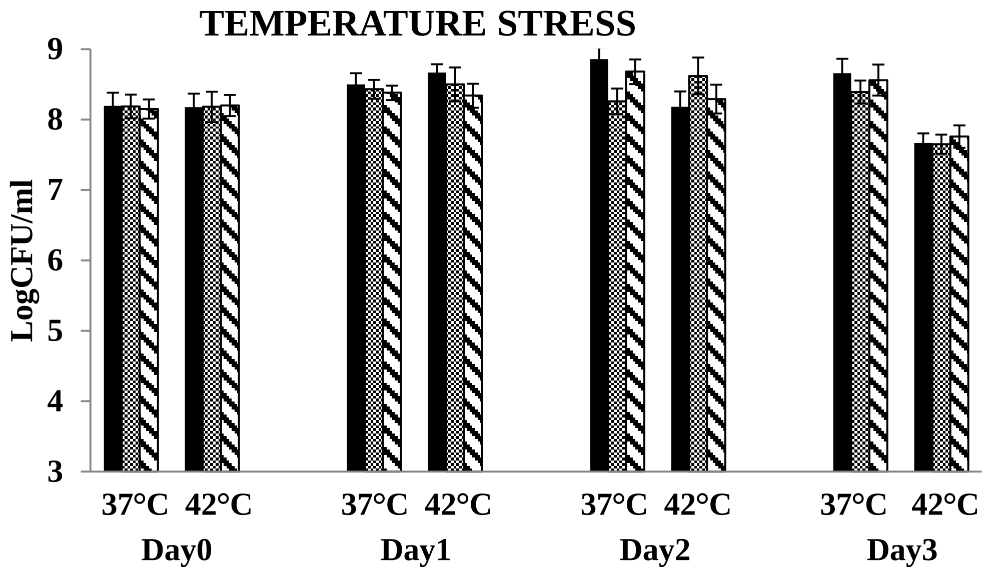
TEMPERATURE STRESS
LogCFU/ml
9
8
7
6
5
4
3
37°C 42°C
Day0
37°C 42°C
Day1
37°C 42°C
Day2
37°C 42°C
Day3

F.

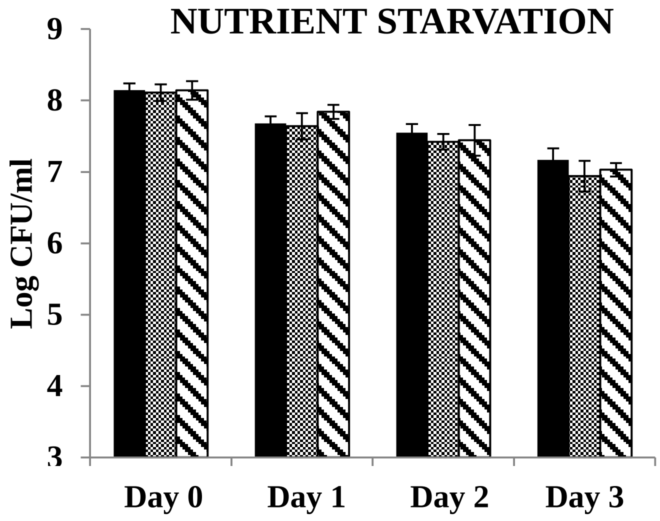
NUTRIENT STARVATION
Log CFU/ml
9
8
7
6
5
4
3
Day 0
Day 1
Day 2
Day 3

Fig S7. Regulation of M*parDE2* locus in *M. smegmatis* under stress conditions.

MS8, MS9 and MS12 cells were grown to different growth phases and subjected to different stress conditions. CFU counts were determined before and after stress treatment. **(A),** To rule out plasmid instability**,** vitamin C treated cultures were taken and plasmid profile was checked by PCR using operon specific (*parD2*-F and *parE2Δ10*-R) and kanamycin specific primers (*aph*-F and *aph*-R, see Fig. S3). Both the MS8 (*parDE2* operon) and MS9 (truncated *parDE2* operon) strains showed similar intensity of the amplified product whereas no amplification was observed in the MS12 vector control or the host *M. smegmatis* mc2 cells (negative control). With kanamycin specific primers all the three strains produced amplicons of similar intensity, while no amplification in the host strain. **(B)**, The cultures were incubated in hypoxic condition and analysed at 0, 5 and 10 days and CFUs were counted. **(C)**, for iron depletion studies, cells were grown in RH defined medium (lacking FeSO4.7H2O) containing 100 µM deferoxamine mesylate salt and viability was monitored for 5 days. **(D)**, acid stress was applied by growing cells in 7H9 medium of pH 4.0. The data presented are mean ± SE of triplicate wells and are representative of three individual experiments.. **(E)**, for heat stress analysis, cells were grown at 42°C and cell viability was monitored for 72 h. (**F),** for nutrient starvation cultures were grown in sterile 1XPBS (pH 6.6) and growth and viability was monitored up to 10 days
